# Functional characterisation of the amyotrophic lateral sclerosis risk locus *GPX3/TNIP1*

**DOI:** 10.1186/s13073-021-01006-6

**Published:** 2022-01-19

**Authors:** Restuadi Restuadi, Frederik J. Steyn, Edor Kabashi, Shyuan T. Ngo, Fei-Fei Cheng, Marta F. Nabais, Mike J. Thompson, Ting Qi, Yang Wu, Anjali K. Henders, Leanne Wallace, Chris R. Bye, Bradley J. Turner, Laura Ziser, Susan Mathers, Pamela A. McCombe, Merrilee Needham, David Schultz, Matthew C. Kiernan, Wouter van Rheenen, Leonard H. van den Berg, Jan H. Veldink, Roel Ophoff, Alexander Gusev, Noah Zaitlen, Allan F. McRae, Robert D. Henderson, Naomi R. Wray, Jean Giacomotto, Fleur C. Garton

**Affiliations:** 1 Institute for Molecular Bioscience, The University of Queensland, QLD, Brisbane, 4072 Australia; 2 School of Biomedical Sciences, The University of Queensland, QLD, Brisbane, 4072 Australia; 3 Department of Neurology, Royal Brisbane and Women’s Hospital, QLD, Brisbane, 4029 Australia; 4 Centre for Clinical Research, The University of Queensland, QLD, Brisbane, 4019 Australia; 5 Imagine Institute, Institut National de la Santé et de la Recherche Médicale (INSERM) Unité 1163, Paris Descartes Université, 75015 Paris, France; 6 Sorbonne Université, Université Pierre et Marie Curie (UPMC), Université de Paris 06, INSERM Unité 1127, Centre National de la Recherche Scientifique (CNRS) Unité Mixte de Recherche 7225, Institut du Cerveau et de la Moelle Épinière (ICM), 75013 Paris, France; 7 Queensland Brain Institute, The University of Queensland, QLD, Brisbane, 4072 Australia; 8 Australian Institute for Bioengineering and Nanotechnology, The University of Queensland, QLD, Brisbane, 4072 Australia; 9 University of Exeter Medical School, RILD Building, RD&E Hospital Wonford, Barrack Road, Exeter, EX2 5DW UK; 10 Department of Computer Science, University of California Los Angeles, Los Angeles, CA USA; 11 Department of Bioinformatics, University of California Los Angeles, Los Angeles, CA USA; 12 Florey Institute for Neuroscience and Mental Health, University of Melbourne, Melbourne, VIC 3052 Australia; 13 Calvary Health Care Bethlehem, Parkdale, VIC 3195 Australia; 14 Fiona Stanley Hospital, Perth, WA 6150 Australia; 15 Notre Dame University, Fremantle, WA 6160 Australia; 16 Institute for Immunology and Infectious Diseases, Murdoch University, Perth, WA 6150 Australia; 17 Department of Neurology, Flinders Medical Centre, Bedford Park, SA 5042 Australia; 18 Brain & Mind Centre, University of Sydney, Institute of Clinical Neurosciences, Royal Prince Alfred Hospital, Sydney, NSW 2006 Australia; 19 Department of Neurology, University Medical Center Utrecht Brain Center, Utrecht University, Utrecht, The Netherlands; 20 Department of Medical Oncology, Dana-Farber Cancer Institute and Harvard Medical School, Boston, MA USA; 21 Division of Genetics, Brigham and Women’s Hospital, Boston, MA USA; 22 Department of Neurology, University of California Los Angeles, Los Angeles, CA 90095 USA; 23 Department of Medicine, University of California San Francisco, San Francisco, CA 94158 USA; 24 Queensland Centre for Mental Health Research, West Moreton Hospital and Health Service, Wacol, QLD 4076 Australia

**Keywords:** Motor neurone disease, MND, Genome-wide association study, Computational biology, Zebrafish, Neurodegenerative diseases, Quantitative trait loci, Genes, Regulator, Disease progression

## Abstract

**Background:**

Amyotrophic lateral sclerosis (ALS) is a complex, late-onset, neurodegenerative disease with a genetic contribution to disease liability. Genome-wide association studies (GWAS) have identified ten risk loci to date, including the *TNIP1*/*GPX3* locus on chromosome five. Given association analysis data alone cannot determine the most plausible risk gene for this locus, we undertook a comprehensive suite of in silico, in vivo and in vitro studies to address this.

**Methods:**

The Functional Mapping and Annotation (FUMA) pipeline and five tools (conditional and joint analysis (GCTA-COJO), Stratified Linkage Disequilibrium Score Regression (S-LDSC), Polygenic Priority Scoring (PoPS), Summary-based Mendelian Randomisation (SMR-HEIDI) and transcriptome-wide association study (TWAS) analyses) were used to perform bioinformatic integration of GWAS data (*N*_cases_ = 20,806, *N*_controls_ = 59,804) with ‘omics reference datasets including the blood (eQTLgen consortium *N* = 31,684) and brain (*N* = 2581). This was followed up by specific expression studies in ALS case-control cohorts (microarray *N*_total_ = 942, protein *N*_total_ = 300) and gene knockdown (KD) studies of human neuronal iPSC cells and zebrafish-morpholinos (MO).

**Results:**

SMR analyses implicated both *TNIP1* and *GPX3* (*p* < 1.15 × 10^−6^), but there was no simple SNP/expression relationship. Integrating multiple datasets using PoPS supported *GPX3* but not *TNIP1*. In vivo expression analyses from blood in ALS cases identified that lower *GPX3* expression correlated with a more progressed disease (ALS functional rating score, *p* = 5.5 × 10^−3^, adjusted *R*^2^ = 0.042, *B*_effect_ = 27.4 ± 13.3 ng/ml/ALSFRS unit) with microarray and protein data suggesting lower expression with risk allele (recessive model *p* = 0.06, *p* = 0.02 respectively). Validation in vivo indicated *gpx3* KD caused significant motor deficits in zebrafish-MO (mean difference vs. control ± 95% CI, vs. control, swim distance = 112 ± 28 mm, time = 1.29 ± 0.59 s, speed = 32.0 ± 2.53 mm/s, respectively, *p* for all < 0.0001), which were rescued with *gpx3* expression, with no phenotype identified with *tnip1* KD or *gpx3* overexpression.

**Conclusions:**

These results support *GPX3* as a lead ALS risk gene in this locus, with more data needed to confirm/reject a role for *TNIP1*. This has implications for understanding disease mechanisms (*GPX3* acts in the same pathway as *SOD1*, a well-established ALS-associated gene) and identifying new therapeutic approaches. Few previous examples of in-depth investigations of risk loci in ALS exist and a similar approach could be applied to investigate future expected GWAS findings.

**Supplementary Information:**

The online version contains supplementary material available at 10.1186/s13073-021-01006-6.

## Background

The genetic contribution to the risk of the lower and upper motor neurone degenerative disease amyotrophic lateral sclerosis (ALS) is complex, with evidence for both Mendelian and non-Mendelian inheritance patterns [1]. Known mutations in 16 genes (implicated with unequivocal evidence [1]) are found in up to 15% of cases with ALS and its ALS-overlapping syndromes. For the remaining cases, genome-wide association studies (GWAS) and heritability estimates (*h*^2^ = 0.43) [2] provide evidence to support a polygenic contribution to genetic liability. Despite the likely polygenic genetic liability for the majority of cases with ALS, the current reported GWAS SNP-based heritability estimates for ALS are relatively low (*h*^2^_SNP_ = 0.018–0.08, range across studies) [3, 4] compared to other common CNS diseases (for example 0.26 for schizophrenia) [5] or neurological disorders (for example 0.23 for Parkinson’s disease) [6]. This could indicate that DNA variants not tagged by common SNPs are more important for ALS than for other diseases (i.e. a contribution of rare variants) but may also reflect recognised technical artefacts of ALS GWAS cohorts, i.e. case-only or control-only samples could weaken the real genetic signal through the stringent quality control (QC) process that must be applied to such data.

Nonetheless, the majority of ALS cases are expected to carry a portfolio of risk variants [3, 4, 7–9]. This portfolio remains relatively unknown with just 10 genomic regions identified through the three largest GWAS to date [3, 9, 10] (represented by their most significantly associated SNPs and closest gene body): rs3849943 (*C9orf72*), rs75087725 (*C21orf2)*, rs117027576 (*KIF5A*), rs616147 (*MOBP*), rs10139154 (*SCFD1*), rs34517613 (*SARM1*), rs74654358 (*TBK1*), rs12608932 (*UNC13A*), rs58854276 (*ZDHHC6*) [11] and a region we initially identified, rs10463311 (*GPX3/TNIP1*) [4] (Additional file 1: Table S1). Exome sequencing and rare-variant burden testing have linked three lead SNP variants to their nearest gene (*C21orf2*, *KIF5A*, *TBK1*) [3, 9] while expression data has implicated five loci (Additional file 1: Table S1).

We had previously identified links with known ALS genes on each side of the lead variant rs10463311 (*GPX3/TNIP1*) on chromosome five [4]. *TNIP1* (encoding TNFAIP3 Interacting Protein 1) has the closest gene body to this sentinel SNP, but the second closest transcriptional start site (TSS) ~ 56 kb downstream (as it is on the reverse strand). Briefly, *TNIP1* is a nuclear factor kappa-B (NF-κB) that interacts with proteins encoded by two known ALS genes *OPTN* and *TBK1* [12, 13]. Upstream of the sentinel SNP is *GPX3*, which encodes *g*lutathione peroxidase 3. The TSS for *GPX3* is located ~ 10 kb upstream (forward strand), and it is a well-known glutathione peroxidase that performs antioxidant functions linked with the most recognised ALS gene, *SOD1* [14]. To identify and understand putative mechanistic contributions of the implicated genes, it is critical to fine-map GWAS-associated loci. Unclear is which method/s are most likely to identify the causal gene from ALS GWAS loci or other neurological conditions.

Bioinformatic methods can quickly and cost-effectively integrate SNP-disease trait GWAS results with SNP-functional trait annotations. Harnessing GWAS results with relevant datasets (genome-wide) can allow the search space to be narrowed to a single region/gene that is likely to contribute to the association. Complementing this with subsequent analyses specific to the disease can help determine mechanisms that could be relevant to target therapeutically [15–17].

Here, a set of complementary bioinformatic approaches implicate both *GPX3* and *TNIP1* genes in the context of ALS risk, with straightforward follow-up approaches that could investigate future ALS GWAS loci. Our in vivo (disease cohorts and a zebrafish model) but not in vitro (human motor neurons) studies offer support for targeting *GPX3* in future studies*.*

## Methods

### In silico annotation of GWAS summary data

**Fig. 1:**
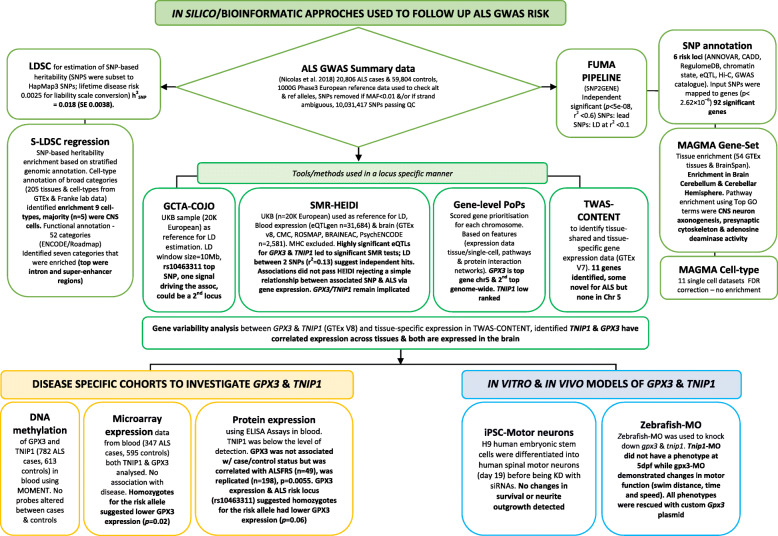
Summary of the in silico, in vitro and in vivo follow-up of ALS GWAS findings. Causal genes/genomic loci implicated by a SNP association are not necessarily the closest (empirical estimate of those that are ~ 30%, Zhu et al. [18]) and the flowchart provides a stepped example on how this paper integrated complementary bioinformatic methods on GWAS summary statistics to prioritise *GPX3* and *TNIP1* for follow-up in disease cohorts and in vitro (human spinal motor neurons) and in vivo (zebrafish-MO) models. Relevant bioinformatic tools and processes used in this paper are described [5, 18–23]. GWAS genome-wide association study, *h*^2^_SNP_ proportion of heritability explained by SNPs, FUMA Functional Mapping and Annotation pipeline, S-LDSC Stratified Linkage Disequilibrium Score regression, GCTA-COJO conditional and joint analysis, SMR-HEIDI Summary Mendelian Randomisation and the HEterogeneity In Dependent Instruments test, TWAS-CONTENT transcriptome-wide association study and context-specific genetics test, PoPS Polygenic Priority Score, siRNA short interfering RNA, iPSC induced pluripotent stem cells, Zebrafish-MO zebrafish morpholino, UKB UK-biobank, chr chromosome

The FUMA pipeline and five complementary tools (GCTA-COJO, S-LDSC, PoPS, SMR- HEIDI, TWAS) were used to perform post-GWAS analysis using the most recent published ALS summary data (*n* = 20,806 cases, *n* = 59,804 controls) [9] (Fig. 1). These analyses use the full GWAS summary data (unless specified) to provide a genome-wide result overview prior to analyses on the *GPX3/TNIP1* locus in chromosome five [4, 9]. The GWAS summary statistics [9] (i.e. SNP ID, allele frequency, association effect size and *p*-value) already controlled for variables, such as sex and ancestry, were run through a standard quality control (QC) pipeline. Briefly, the European 1000 Genome phase 3 reference data was used to ensure that the reference and alternative alleles were correct, SNPs were removed with MAF < 0.01 and/or if strand-ambiguous (AT or GC alleles). This process resulted in 10,031,417 SNPs passing QC, ready for post-GWAS analysis. SNP-based heritability was estimated by using LD-score regression [5]. This was applied to GWAS summary statistics to estimate the contribution of common genetic variants to variation in the liability of ALS [24]. Lifetime disease risk of 0.0025 was used in the conversion of the estimate to the liability scale.

#### FUMA

The FUMA (v1.3.5d) pipeline [19] annotated the GWAS results based on positional and functional information of SNPs. Briefly, significantly associated SNPs are characterised as risk loci by incorporating linkage disequilibrium (LD) structure to prioritise genes that are likely to be involved in ALS (SNP2GENE). Functionally annotated SNPs are mapped to genes based on three strategies, positional mapping (functional consequences on genes), expression (expression quantitative trait loci (eQTLs)) and chromatin interactions of phenotype-relevant tissue types (eQTL and chromatin interaction mapping). GWAS summary statistics were uploaded and run using the default SNP2GENE job settings using available biological data repositories and tools. Available data repositories used in FUMA (and other tools) have been through prior quality control pipelines to adjust and control for variables such as sex and age. To identify independent significant SNPs, a *p*-value threshold of < 5 × 10^−8^ was used and an LD *r*^2^ of < 0.6 with other more significantly associated SNPs. Within this pool of independent SNPs, lead SNPs were defined as those most highly associated and independent from other lead SNPs (LD of *r*^2^ < 0.1). The lead SNPs and those in LD with them were annotated as risk loci (a 250 kb window, *r*^2^ ≥ 0.6). The 1000 genomes phase 3 data [25] were used as the LD reference (all ancestries as recommended [19]). The MAGMA gene-set analysis [26] was used to identify significant genes (from a total of 19,290 genes from Ensemble v92) (Bonferroni-adjusted *p*-value of 0.05/19,290 = 2.6 × 10^−6^). These were used for MAGMA pathway analysis (using the full distribution of *p*-values and 5917 GO terms obtained from MsigDBv6.2, and a Bonferroni-adjusted *p*-value of 8.4 × 10^−6^) [27]. Gene-based test association results were used to identify relevant tissue enrichment (*n* = 54 tissues GTEx v8) [28] and BrainSpan [29].

We also tested the relationships between ALS GWAS associations and the single-cell gene expression profiles and associations. The analysis was based on a regression model and a one-sided test (*βE* > 0) [20] using 11 single-cell datasets (Additional file 2: Supplementary Methods) with an FDR multiple correction applied.

#### S-LDSC

Stratified Linkage Disequilibrium Score Regression (S-LDSC) [21, 22] was applied to the ALS GWAS summary statistics [9] to determine the genomic functional annotations most relevant to the common variant association signal. The GWAS summary statistics were subset to HapMap3 SNPs and used as the input (S-LDSC default). For functional annotations, SNP-based heritability was partitioned into 52 categories in S-LDSC based on reference data from ENCODE [30] and Roadmap Epigenomics Consortium data [31], including elements such as UCSC, UTRs, promoters, intronic regions and conserved regions. For the S-LDSC cell-type annotation analysis, SNP-based heritability was partitioned to compare across cell-specific annotations, including GTEx [32] and Franke lab data [33] set comprising 205 tissues and cell types (categorised to nine broad tissues categories for visualisation). We used the LDSC Python command line tool [5] to run the analysis and an FDR-corrected *p*-value of < 0.05 to determine significance.

#### GCTA-COJO

To determine if GWAS associations at the chromosome 5 locus could represent more than one causal variant, we performed a conditional and joint multiple SNP analysis implemented in GCTA software (GCTA-COJO) analysis [34]. The QC’ed ALS GWAS summary statistics were used with LD reference from imputed-genotyping data of UK Biobank (20,000 individuals randomly sampled of European descent). We used the default settings limited to chromosome 5 (using --chr 5 option) to identify both the lead SNP and the top 10 independent SNPs. Then, to detect if there was evidence for collinearity, we conditioned on the lead SNP. Using a similar method, we examined the top eQTL SNP for *GPX3/TNIP1* from eQTLgen summary statistics (rs12518386) by conditioning on the lead SNP and comparing the top eQTL SNP effects and *p*-values. If the effects become very small or *p*-values become less significant, this means the top eQTL SNP effect is driven, at least in part by LD with the lead SNP, while the opposite result is a sign/possibility of independent effects. To validate/confirm the result, we then conditioned on top eQTLgen SNP.

#### SMR-HEIDI

We conducted a Summary data-based Mendelian Randomisation **(**SMR) analysis [18] to investigate if ALS SNP associations were mediated through expression. This method applies a Mendelian Randomisation framework to infer causality by testing the association between GWAS and expression quantitative trait loci (eQTL) summary statistics. We also apply the HEterogeneity In Dependent Instrument (HEIDI) test to distinguish, where possible given the data, causality (or pleiotropy) from linkage [18]. When an SMR association passes the HEIDI test to indicate low heterogeneity (*p* > 0.05), the data are consistent with the ALS-associated SNP reflecting differences in gene expression of the risk and protective alleles. For the analysis, the QC’ed ALS GWAS summary statistics were used with LD reference from imputed-genotyping data of UK Biobank (20,000 individuals randomly sampled of European descent). For the expression data, we used eQTL data with the largest sample size (blood gene expression eQTLGen, *n* = 31,684) [35] as well as relevant ALS tissues (brain, cell-type annotation results) [36]. The brain samples had been previously meta-analysed by Qi et al. [36] (GTEx, version 7.35 [28], the CommonMind Consortium [37], the Religious Orders Study and Memory and Aging Project (ROSMAP) project [38], Brain eQTL Almanac project (Braineac; 10 brain regions) [39]) (effective sample size of *n* = 1194) and the PsychENCODE brain prefrontal cortex eQTL data (*n* = 1387) (SCZ, bipolar disorder and autism spectrum disorder) [40] (*N*_total_ = 2581). The large sample size (eQTLgen) is best powered for detection of QTLs [32] and can be used as a proxy, given top cis-eQTLS are correlated across tissues [32] including those between independent brain and blood samples [36]. To ensure relevant tissue-specific eQTLs for ALS are still considered, we use eQTL data from brain samples (despite their smaller sample sizes and hence reduced power for detection of eQTLs (compared to the blood eQTLgen data)). For all of the eQTL data, we excluded cis-eQTL with MAF < 0.01 and the MHC region (to avoid misinterpretation due to the LD complexity). We chose the significant probes from SMR analysis with stringent Bonferroni-corrected threshold for SMR *p*-value (0.05/number of probes) and > 0.05 as the HEIDI test *p*-value threshold. Given blood eQTL data were used as a proxy for more ALS-relevant tissues due to its large sample size, significant SMR findings were followed up in the brain meta-analyses using Bonferroni threshold corrected for significant findings (rather than genome-wide probe number). The use of both SMR and HEIDI methods is ideal for prioritising loci for functional follow-up if studies are sufficiently powered and conservative thresholds are applied.

#### TWAS and TWAS-CONTENT

Transcriptome-wide association studies (TWAS) can provide insight into gene-trait associations by summarising the effects of eQTLs into a single, powerful predictor of gene expression [41]. First, we built genetic models of gene expression (training an elastic net on a tissue-by-tissue basis) (GTEx v7 consortium) [28] before running the gene-tissue weights and ALS summary statistics [9] through TWAS. In addition to the original TWAS approach, we performed a similar analysis using CONtexT spEcific geNeTics (CONTENT) [42] based on the methodology and decomposition of a previous work by Lu et al. [43]. CONTENT uses individual-level data to first decompose a gene-tissue’s expression into both a tissue-shared component as well as a tissue-specific component, then trains an elastic net on each component separately (Additional file 2: Supplementary Methods). CONTENT then builds a final predictor—termed the CONTENT “full” model—which combines both predicted components of the expression. Consequently, CONTENT can discover for a given eGene a component that is shared across all tissues, components that are specific to tissues, and/or a predictor that includes both the tissue-shared and tissue-specific components. As there are multiple tests to determine whether a gene contains a heritable component, CONTENT leverages a hierarchical FDR set at 0.05 [44] to conservatively correct for multiple testing. Identifying tissue-specific eQTLs may provide additional insight relevant to disease phenotypes rather than eQTLs that affect expression across multiple tissues.

#### PoPS

We utilised a similarity-based gene prioritisation method, Polygenic Priority Score (PoPS) [23], to identify top gene candidates in each chromosome. Using the ALS summary GWAS [9], PoPS excludes the locus of interest and uses marginal feature selection to weight those considered relevant. Briefly, it performs a gene-based association using MAGMA [26] and then performs an enrichment analysis for each gene feature and those that are nominally significant are retained (*p* < 0.05). The features are created from expression data, single-cell datasets, pathway data and protein-protein interaction networks (many of which are not jointly considered in other tools). Joint enrichment (of the selected features) is computed using a generalised least squares (GLS) regression model which also includes a matrix of gene-level covariates such as gene length. A leave one chromosome out (LOCO) framework is then used to compute a polygenic priority score for each gene, per chromosome, by multiplying its row vector of gene features. In this manner, PoPS provides a score for each gene (independent of the GWAS data on the chromosome where the gene is located) to prioritise a gene in the locus of interest for each chromosome.

### Human samples

To examine *GPX3* and *TNIP1* expression in the context of disease, Australian ALS cases and controls were recruited (2016–2019) with written consent obtained from all individuals (discovery cohort *N*_cases_ = 50, *N*_controls_ = 50; replication cohort *N*_cases_ = 200, *N*_controls_ = 28) (Table 1). The discovery sample cohort was from a single site (The Royal Brisbane and Women’s Hospital (Brisbane), while the replication cohort (independent samples) included three additional Australian sites, Flinders University (Adelaide), Fiona Stanley Hospital (Perth) and Calvary Health Care Bethlehem (Melbourne). Each site had study approval from their local Human Research Ethics Committee (HREC). Clinical data were recorded (research nurses/neurologists) on a single secure server that included the generation of a de-identified subject ID. This ID was used during subsequent processing at The University of Queensland. ALS cases fulfilled the revised El Escorial criteria for possible, probable (lab-supported) or definite ALS. Retrospective analysis using the Gold Coast diagnostic criteria [45] was also applied, and a subset analysis removing subjects not meeting the criteria was applied (Additional file 2: Supplementary Methods). Control subjects were unrelated, age-matched individuals free of neuromuscular diseases, recruited as either partners or friends of patients with ALS or community volunteers. Available demographic and clinical data was matched with the subject and collection ID (Table 1). All participants were confirmed to be of European ancestry with genotyping data when possible [3, 4] with previously published data utilised for genotype [3, 4] and methylation [46] analyses.

**Table 1:** Clinical details of the cohort utilised for the ELISA assays

Cohort (location)	Preliminary (discovery cohort) Australian (Brisbane-based)		Independent replication cohort Australian (Australia-wide*)	
Sample	ALS cases	Controls	ALS cases	Controls
**Number**	50	50	200	28
**Age (yrs, ± 95% CI)**	61 ± 2.3	60 ± 1.9	62.7 ± 1.6	52.9 ± 5.4
**Sex (F/M)**	13/37	18/32	59/141	15/12
**BMI**	25.7 ± 1.0	26.8 ± 1.2	NA	NA
**Smoker (ever)**	Yes = 20 No = 30 NA = 0	NA	Yes = 15 No = 79 NA = 106	NA
**ALSFRS-R**	38 ± 1.2	NA	32.9 ± 1.2	NA
**Age at onset**	59.0 ± 2.4	NA	60.3 ± 1.8	NA
**Age at diagnosis**	60.7 ± 2.5	NA	61.5 ± 1.9	NA
**ALS onset site**	B = 11 (22%) UL = 13 (26%) LL = 21 (42%) Other = 5 (10%)	NA	B = 44 (26%) UL = 51 (30%) LL = 70 (41%) Other = 6 (4%) NA = 29	NA
**ALS type**	Classic = 30 UMN = 8 LMN = 8 Other = 4	NA	Classic = 97 UMN = 8 LMN = 13 Other = 81	NA
**Family history**	0	NA	22 (11%)	NA
**FVC (seated)**	3.6 ± 0.3	NA	NA	NA
**NIV**	3	NA	NA	NA
**PEG**	5	NA	NA	NA
**Riluzole**	25/50	NA	NA	NA
**rs10463311** **Genotype** ***n*** **(%)**	TT = 23 (50%) TC = 17 (37%) CC = 6 (13%) NA = 4	TT = 26 (59%) TC = 15 (34%) CC = 3 (7%) NA = 6	TT = 114 (62%) TC = 59 (32%) CC = 11 (6%) NA = 16	TT = 12 (44%) TC = 14 (52%) CC = 1 (4%) NA = 1
**Months between onset and assessment**	22.4 ± 5.1	NA	33.5 ± 4.5	NA
**Months between diagnosis and assessment**	9.3 ± 3.1	NA	22.92 ± 5.9	NA
**Comorbidity**	32/50	30/50	NA	NA
**GPX3 level (ng/ml)**	1742.2 ± 350.3	1908.0 ± 315.0	4907.8 ± 225.1	5368.4 ± 599.9
**TNIP1 level**	Not detectable	Not detectable	NA	NA
**Days between blood collection and plasma extraction**	0 ± 0	0 ± 0	1.7 ± 0.1	0.9 ± 0.3
**Visit to the clinic which the sample was collected**	NA	NA	1.35 ± 0.1	NA
**Rate of progression**^κ^	NA	NA	0.71 ± 0.09	NA

*F* female, *M* male, *BMI* body mass index, *ALSFRS-R* Amyotrophic Lateral Sclerosis Functional Rating Score – Revised (range 0–48 (48 = no physical disability)), *FVC* forced vital capacity, *NIV* non-invasive ventilation, *PEG* percutaneous endoscopic gastrostomy. *Samples were collected from four clinics, *κ* = change in ALSFRS per month since onset ((48-ALFRS at visit)/(months between onset and visit date)) (*n* = 128, Additional file 2), ±error indicates 95% confidence interval

### mRNA in ALS cases and controls

Microarray expression data from blood with matched genotyping data from a Netherlands cohort (*n* = 942, *N*_cases_ = 347, *N*_controls_ = 595) were examined for evidence of changes in expression with reference to the risk allele [47]. Briefly, mRNA was isolated and purified using PAXgene tubes and extracted according to the instructions (QIAGEN RNA extraction kit). Samples were hybridised to two different platforms (HumanHT-12 v3 and v4 BeadChips) according to the manufacturer’s protocol (Illumina, Inc., San Diego, CA, USA). Standard normalisation (using overlapping probes) and QC were carried out including sex checks. Surrogate variable analysis was used to calculate residuals to correct for known and unknown technical effects in a linear regression model [48]. All associations included covariates to correct for batch effects and sex. *GPX3* and *TNIP1* gene expression were examined between cases and controls with respect to the risk variant.

### GPX3 and TNIP1 protein expression in blood plasma

GPX3 and TNIP1 expression were measured in plasma in ALS cases (*N* = 50) and controls (*N* = 50) using commercially available sandwich enzyme-linked immunosorbent assays (ELISA). GPX3 expression was subsequently measured in a set of independent samples (replication cohort, *N*_cases_ = 200, *N*_controls_ = 28). All plasma samples were extracted from venous blood collected in an EDTA tube. These were stored at room temperature during transportation and then centrifuged at 1000*g*. Plasma was frozen in 500-μl aliquots and stored in a −80 freezer before being thawed on ice before use. TNIP1 activity measured using the ELISA kit (MyBiosource, Inc, San Diego, cat:MBS925301) was not detectable, and no standard curve could be generated despite running plasma at the highest possible concentration. GPX3 activity was detected in plasma samples, and after a standard curve optimisation following kit instructions (Adipogen, Liestal Switzerland, cat: AG-45A-0020YEK-KI01), case and control plasma was diluted at 1 in 200 (ELISA Buffer 1X) and run in duplicate for the assay following kit instructions (Additional file 2: Supplementary Methods). As described above, this was carried out in two batches, a preliminary discovery cohort (*N*_total_ = 100) and an independent replication cohort (*N*_total_ = 228) (Table 1). All cases had a time-matched ALSFRS score recorded. In the discovery cohort, fifty cases and fifty controls were used to measure GPX3. These blood samples had been collected in EDTA tubes, spun and frozen within 24 h. The replication set consisted of a larger sample size in which blood samples had been collected, spun and plasma frozen between 0 and 4 days (200 cases and 28 plate controls) (Table 1). Methods for GPX3 detection were identical in each set, and cases and controls were randomised across plates. Fluorescence was directly proportional to the concentration of GPX3 in the samples and was calculated based on the plate standard. Data analysis was carried out in R. Preliminary analyses assessed covariates such as age, sex, BMI and days post-collection (0–4) to determine if they had an effect on GPX3 expression (only sex had a detected effect and was included as a covariate). To assess the risk allele on GPX3 expression, available genotype data was matched with samples in both the discovery and replication cohorts. To meta-analyse the results using both additive and recessive models, GPX3 levels were standardised (mean of 0 and standard deviation of 1) in each experiment.

Just under half the participants with ALS provided blood samples and clinical data at multiple clinic visits allowing a preliminary longitudinal analysis to be conducted (*N*_cases_ = 89, *N*_measurements_ = 224 (2–5 visits)). Change in ALSFRS-R (a clinical questionnaire assessing functional disability and extent of neuronal loss with scale 48 to 0 (48 = indicating normal physical function and no disability)) uses months since diagnosis and months since first visit as the dependent variable in a linear regression on GPX3 level. While the date of the first symptom and diagnosis date were both available, we used the latter for consistency. Diagnosis is provided by a neurologist and is reliable and memorable while symptom onset is reliant upon a subjective report of symptoms, i.e. the decision of which symptoms represent the onset of disease and so can be more variable. Months since the first visit was used to visualise the data as a few subjects joined the study quite late (~ 50 months post-diagnosis), acknowledging that long survival is a likely ascertainment bias in prevalent ALS samples.

### *GPX3* and *TNIP1* DNA methylation

A subset of the Australian ALS case-control cohort (*N*_cases_ = 782, *N*_controls_ = 613) had data generated from Illumina 450k arrays. These had been previously analysed in a methylome-wide association study with standard quality control and covariates accounted for (i.e. batch effects, cell type, age, sex) [46]. We thus inspected the DNA methylation levels of probes annotated to *GPX3* and *TNIP1* [49] and their corresponding association summary statistics noting that non-variable probes (s.d. < 0.02) were excluded.

### In vitro knockdown of *GPX3* and *TNIP1* in human motor neurons

To test if cell GPX3 or TNIP1 are required for the development and survival of human spinal motor neurons, siRNAs were targeted to these genes. Briefly, H9 human embryonic stem cells (WA09 line, RRID:CVCL_9773, WiCell Research Institute) [50] were differentiated [51] with modifications as previously described [52]. At day 19 of the differentiation, motor neurons were co-transfected with Hb9-GFP reporter constructs and target or scrambled siRNAs (human siRNA Oligo Duplex, locus ID 2878 or 10318, Origene) as described in detail previously [52]. After 48 h, the motor neurons were harvested for gene expression, or morphological and viability analysis using *n* = 3 independent differentiations (normalised to Hb9-GFP only transfected cells). Target siRNA knockdown of *GPX3* or *TNIP1* gene expression was confirmed with real-time qPCR as previously described [52] using primers against *GPX3*, *TNIP1* and the housekeeping gene *HPRT1* (Additional file 2: Supplementary Methods). Live human motor neuron cultures were imaged using HB9-GFP fluorescence on a CellDiscoverer 7 microscope (Zeiss) and assessed for motor neuron morphology and defects in neurite outgrowth [53]. Cell viability was measured using the Thiazolyl Blue Tetrazolium Blue (MTT) reduction assay (Sigma). Both these assays have been linked with known ALS/MND genes. Cell viability is impacted by *FUS* [54] and *SOD1* [55] (cell types, SH-SY5Y, NSC-34), and axonal length defects have been identified by KD of full-length *SMN* (to model spinal muscular atrophy) [56]. Quantification of *GPX3* and *TNIP1* gene expression suggested that greater than 50% of the cells were transfected to provide good sensitivity to detect the viability effect. The axon quantification assays were independent of transfection efficiency as only GFP-positive transfected motor neurons were quantified.

### In vivo knockdown of *GPX3* and *TNIP1* expression in zebrafish

To investigate the consequences of lowered *GPX3* or *TNIP1* levels in vivo (as lower gpx3 expression correlated with worse physical function in disease), zebrafish knockdown (*GPX3* and *TNIP1*) experiments were conducted using antisense morpholino oligonucleotides (MO) to block translation of each human orthologue (one of each; *gpx3* and *tnip1*) (similar to methods previously used for investigation of other ALS-associated genes [57]). As a control, we designed and used a 5-base-pair mismatch MO. Oligonucleotides were designed using Gene Tools LLC (Philomath, OR; http://www.gene-tools.com/) (Additional file 2: Supplementary Methods). To define the optimal dosage, titration experiments were performed using five doses (dose range 0.25–1.2 mM) and injected embryos monitored.

Briefly, adult and larval zebrafish (*Danio rerio*) were maintained at a dedicated zebrafish facility (Imagine Institute and ICM, Institut du Cerveau et de la Moelle épinière, Paris, France) and bred according to the National and European Guidelines for Animal Welfare. Wild-type (AB background) and transgenic (Tg(Mnx1:eGFP)) zebrafish embryos were raised at 28 °C in E3 medium supplemented with 0.01 mg/L methylene blue. Antisense morpholino (MO) sequences (Additional file 2: Supplementary Methods) were designed to complementarily bind to *GPX3* and *TNIP1* genomic DNA sequences and encompassed the respective ATG start codon (to block transcription). Blastomeric microinjections were performed at one-cell stage using glass microcapillaries (Sutter Instrument) and a Picospritzer III pressure injector (General Valve Corporation, Fairfield, NJ, USA). Injected embryos were cultivated in standard incubator condition and development observed for 5 days (dissecting microscope and manual observation) before selecting the most appropriate concentration to perform motor testing. The touch-evoked escape response (TEER) assays were performed at 2 dpf (days post-fertilisation) as previously described [58]. Sex was not considered to have an effect at this early stage of development and was not included in the analyses.

#### Zebrafish mRNA injection and MO-rescue and overexpression experiments

To both test the potential pathogenicity of GPX3 overexpression and its ability to rescue/validate the observed GPX3-LOF motor phenotype, we designed a custom-GPX3 mRNA (cstGPX3) with a modification of the first two codons (Additional file 2: Supplementary Methods) to protect the synthetic mRNA from being targeted by the aforementioned GPX3-MO, while maintaining the same protein sequence. The cstGPX3 mRNA was synthesised as described in Additional file 2: Supplementary Methods and then purified and stored at −80 °C prior to the experiments. Injections and titrations were performed as described above with a dose ranging from 50 to 250 pg final. Rescue experiments were performed at a final concentration of 100 ng/μl [58].

## Results

Prior to proposing the follow-up of any ALS risk locus, we conducted a suite of post-GWAS analyses on the most recent ALS GWAS summary statistics [9]. These analyses were not included in that study and thus were carried out here (Fig. 1). This provided support for the hypothesis that the GWAS association signal includes true positive information (i.e. evidence consistent with a CNS/neurological disease basis), despite the low SNP-based heritability that we estimated from these data using LDSC regression (*h*^2^_SNP_ = 0.018, *SE* 0.0038).

### Annotation of full ALS GWAS identifies enrichments consistent with known disease processes

The Functional Mapping and Annotation pipeline (FUMA v1.3.6) detected six significant genomic risk loci associated with ALS (Additional file 1: Table S1-S2). Within these loci, 201 candidate SNPs (*r*^2^> 0.6 (measure of linkage disequilibrium)) and 43 independent (*r*^2^< 0.6) significant SNPs were identified, resulting in 92 prioritised genes (*p* < 2.6 × 10^−6^) (Additional file 1: Tables S2-S5, Additional file 2: Figs. S1-S2). The gene-property analysis (using MAGMA [26]) identified the following top gene ontology pathways “go central nervous system neuron axonogenesis” (category: GO biological processes *p*-value = 2 × 10^−4^), “go presynaptic cytoskeleton” (category: cellular component, *p*-value = 6 × 10^−4^) (disease process [59];) and “go adenosine deaminase activity” (category: molecular function cellular component *p*-value = 5 × 10^−4^) (disease process [60];) (Additional file 1: Table S6).

**Fig. 2:**
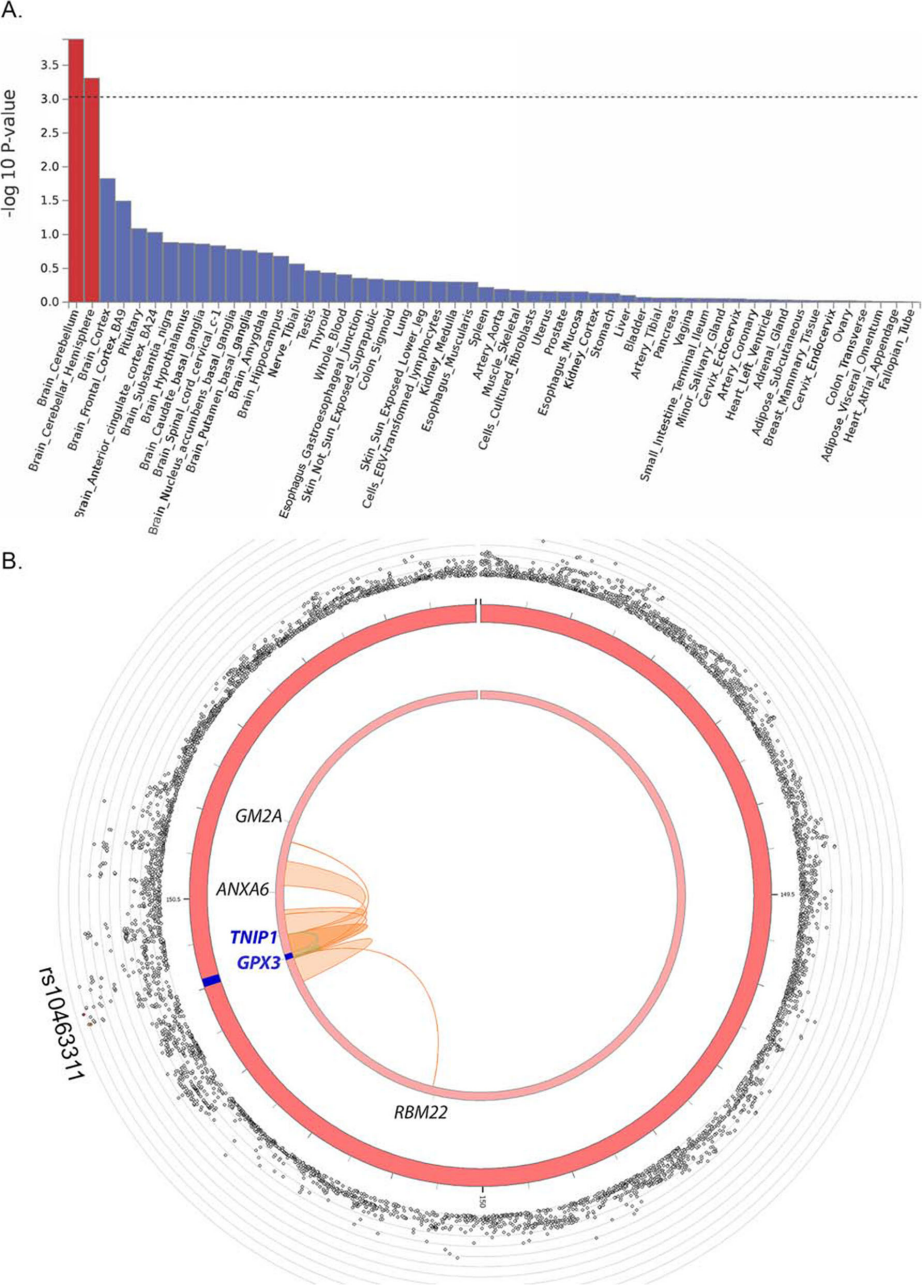
Functional annotation of ALS GWAS. **A** Using the full GWAS summary data, enrichment of association signal is identified in genes expressed in brain tissue using GTEx v8 (*n* = 54 tissues). **B** Circos plot of chromosome 5 with the risk locus in blue (middle circle); outer circle shows SNP associations (grey circles) with -log_10_(*p*-value) on the *Y*-axis. The lead SNP (rs10463311) is labelled, and other SNPs are coloured if they are in LD of the lead SNP (yellow to red, low to high *r*^2^, see Fig. 3 for detail). Inner circle: The mapped genes are labelled black if chromatin interaction is detected (*GM2A*, *ANXA6*, *RBM22*), and blue if both a chromatin interaction and an eQTL is detected (*TNIP1*, *GPX3*)

Stratified LD-score regression (S-LDSC) [5] provided functional annotation and cell-type SNP-based heritability enrichment (i.e. the proportion of SNP-based heritability divided by the proportion of SNPs in the category) estimated from GWAS summary statistics. Seven annotation categories were significantly enriched (FDR-corrected *p*-value of < 0.05) with the two most significant enrichments identified as “Intron” and “Super enhancer” regions (both with 500-bp extension) (Additional file 2: Fig. S3 and Additional file 1: Table S7). S-LDSC analysis using cell-type annotation identified nine cell types with gene expression enrichment. This was dominated by cells in the tissue category of the “Central Nervous System” (6/9) and the most significant cell enrichment corresponded to “Muscle-Skeletal” (Additional file 2: Fig. S4, Additional file 1: Table S8). Functional annotation of the full GWAS summary data similarly identified enrichment in the “Brain Cerebellum” and the “Brain Cerebellar Hemisphere” (*p*-value < 3 × 10^−10^) based on gene expression in 54 tissue types from GTEx v8 (Fig. 2A). We did not identify further fine-scale enrichments specific to age (i.e. Brainspan data, 29 ages/11 developmental stages) or single-cell type (brain, blood and muscle).

### Annotation of the locus on chromosome 5 implicates *GPX3* and *TNIP1*

The GWAS enrichment in genes differentially expressed in the CNS and relevant functional pathways (despite the low overall SNP-based heritability) provided confidence to continue with more in-depth analyses of the chromosome five locus (5:150,401,796 -5:150,410,835). A suite of tools were utilised (Fig. 1) as combining results from different methods yields more robust results [61].

Three candidate SNPs identified in FUMA included the sentinel SNP rs10463311 (*p* = 4 × 10^−8^) and two correlated SNPs rs4958872 (*r*^2^ = 0.62, *p* = 6 × 10^−8^) and rs3828599 (*r*^2^ = 0.64, *p* = 8 × 10^−8^) (Additional file 1: Table S4). Each SNP was in an intronic region with a CADD (PHRED) score (a measure of deleteriousness of single nucleotide variants) that did not indicate any evidence for deleteriousness (scores of 1.03, 0.69 and 4.61, respectively, which are in the bottom 95% of all reference single nucleotide variants in scaled CADD units) (Additional file 1: Table S4). Their RegulomeDB scores (5, 4 and 5, respectively) indicated the locus was associated with transcription factor (TF) binding and/or a DNase peak, and the minimum (and most common) 15-core chromatin state across 127 tissue/cell types suggests that these are typically in areas of open chromatin (Additional file 1: Table S4).

To determine, in a formal statistical framework, whether there is evidence for one or multiple signals in the locus, we applied conditional and joint analysis to the GWAS summary statistics using GCTA-COJO [34]. These analyses (Additional file 1: Table S9-S10) were consistent with just one association signal (rs10463311).

Genes in the region were then prioritised by functional mapping using eQTL (SNPs associated with variation between people in gene expression) and available chromatin interaction data. Five genes were identified. Three genes (*RBM22*, *ANXA6*, *GM2A*) were linked via regional SNP-chromatin interactions (one or two cell types) (Additional file 1: Table S11) but were not linked to altered expression. *GPX3* and *TNIP1* had regional SNP-chromatin interactions in multiple cell types and were also identified as eQTLs in multiple tissues to support them as the lead candidates (Fig. 2B) (Additional file 1: Tables S5 and S11).

### SMR implicates both *GPX3* and *TNIP1* in blood and *GPX3* in the brain

**Fig. 3:**
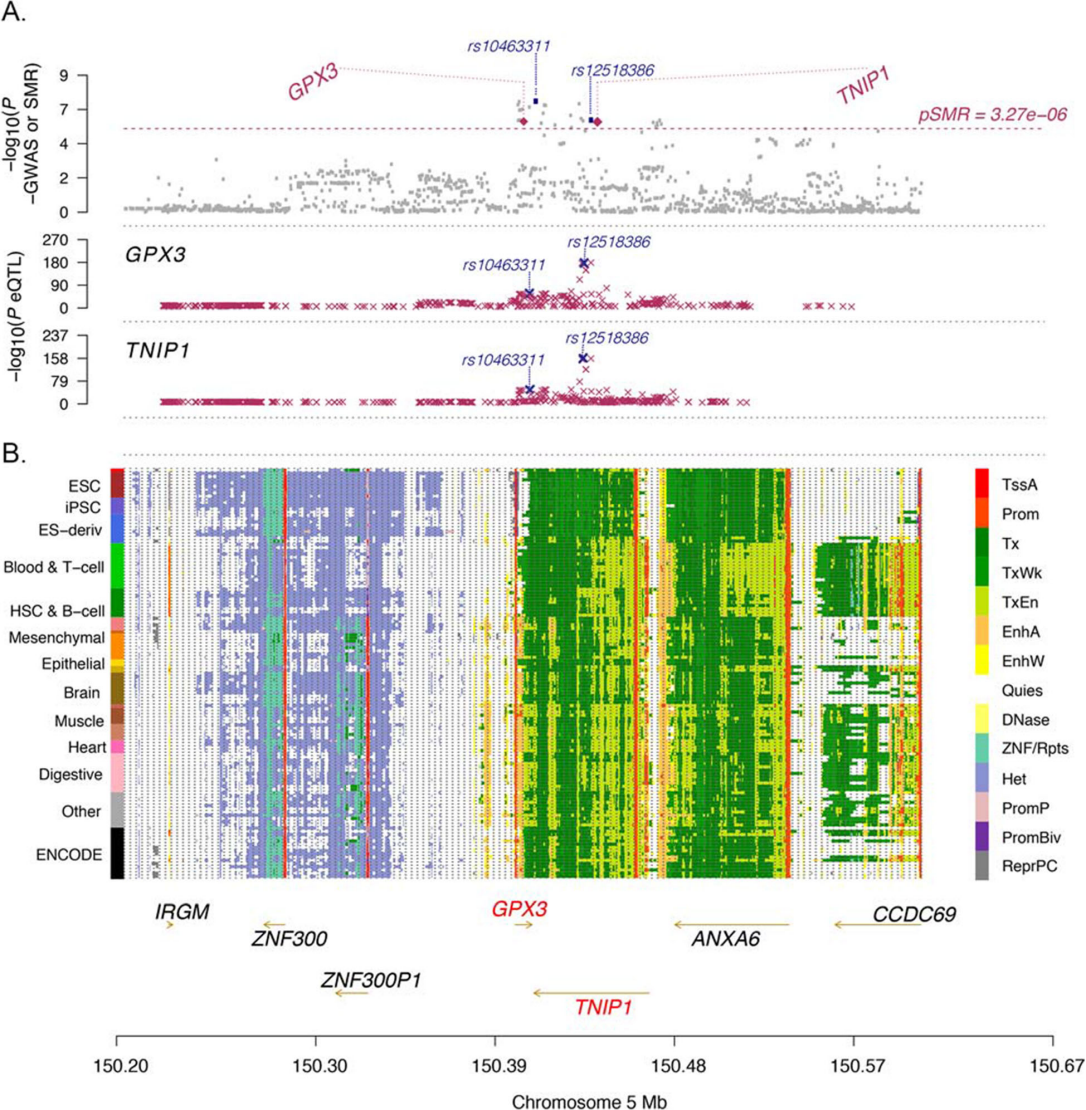
Summary statistics-based Mendelian Randomisation (SMR) analysis identifies *GPX3* and *TNIP1*. Regional map association plot of *GPX3* and *TNIP1* from summary statistics-based SMR analysis. The *x*-axis, chromosome position, is the same in all plots. **A** Grey dots represent the GWAS *p*-values, with the purple diamonds representing the SMR test *p*-values for the two genes (*GPX3* and *TNIP1*) probes that pass the SMR genome-wide significance threshold (dashed line). The purple crosses are the association *p*-values between the SNP and gene probe. The SNP most highly associated with both *GPX3* and *TNIP1* expression is rs12518386 (GWAS association *p*_GWAS_ = 8.33 × 10^−7^, *B*_effect GWAS_ = 0.08, *p*_eQTL:GPX3_ = 1.05 × 10^−171^, *p*_eQTL:TNIP1_ = 2.04 × 10^−163^). The SNP most associated with ALS is rs10463311 (*p*_GWAS_ = 4.00 × 10^−8^, *B*_effect GWAS_ = 0.09, *p*_eQTL:GPX3_ = 4.85 × 10^−18^, *p*_eQTL:TNIP1_ = 1.80 × 10^−36^) (Additional file 1: Table S12). **B** The locus is in a region of open chromatin (each row is a cell type, 13 tissue/cell categories in total on the left) including transcriptional areas and enhancers (coloured legend on the right) between *GPX3* (transcribed on the forward strand) and *TNIP1* (transcribed on the reverse strand)

Summary-based Mendelian Randomisation (SMR) and its methodological partner HEterogeneity In Dependent Instruments (HEIDI) [18] provide a statistical framework to evaluate evidence for whether a SNP-trait (here SNP-ALS) association is being mediated via gene expression through integration with eQTLs identified as associated at the level of genome-wide significance. We used both whole blood and meta-analysed brain eQTL data to carry out SMR analyses. Whole blood eQTL data were used a proxy to detect relevant genes as the sample size (*n* = 31,684 eQTLgen consortium [35]) is much bigger than ALS-relevant tissues (i.e. our brain meta-analyses *N*_total_ = 2581), and it is recognised that many eQTLs are shared across cell types [32, 36]. The SMR analysis identified colocalisation of SNP-trait and eQTL associations in six genes including *C9orf72* (chr9), *GPX3*, *TNIP1* (chr5) and *TRIP11*, *SCFD1* and *RP11-529* (chr14) (Additional file 1: Tables S12-13). Both *GPX3* (*B*_SMR_ = 0.30 ± 0.062, *p* = 1.1 × 10^−6^) and *TNIP1* (*B*_SMR_ = −0.31 ± 0.064, *p* = 1.2 × 10^−6^) (Fig. 3, Additional file 2: Fig. S5) had the same top SNP rs12518386, which was in low LD (*r*^2^ = 0.13) with the lead GWAS SNP rs10463311.

We then used the HEIDI test to consider the pattern of the eQTL SNP associations. This determines if the association signals of SNPs that are physically close to the most significant SMR-association SNP follow a pattern expected by their correlation (LD) structure. The SMR association at rs12518386 did not pass the HEIDI test (*p* < 0.003 (pass threshold > 0.05)) which implies a more complex association pattern than a single SNP relationship (Additional file 1: Table S13). With current data, we cannot exclude that the significant SMR results reflect linkage between the trait-associated SNP rs10463311 and eQTL SNP rs12518386. Hence, larger sample sizes for both ALS GWAS and eQTL are needed to further clarify these results. While this limits our conclusions on whether there is a simple SNP-gene expression relationship, the same genes *GPX3* and *TNIP1* remain implicated in the locus.

The SMR analyses in brain eQTL data combined several consortia in two meta-analyses (PsychENCODE [40] and Brainmeta (CommonMind [37], Braineac [39], GTEx v7 [28] and ROSMAP [38], *n* = 1194–1387)). With much smaller sample sizes, only *C9orf72* was detected using the genome-wide probe-corrected threshold (*B*_SMR_ = 0.07, *p* = 1.77 × 10^−6^) (Additional file 1: Table S13). To examine the correlation between blood and brain eQTLs (many eQTLs are shared across cell types [32, 36] but not all [62]), we examined the genome-wide significant eQTLs in blood and the GWAS risk-allele in the two brain eQTL datasets. Four genes (of six) discovered in blood were further replicated in brain eQTL SMR analysis at the nominal threshold (Bonferroni-corrected *p-*value < 0.0083), indicating that these genes (*GPX3*, *C9orf72*, *SCFD1*, *RP11-529H20.6*) are also likely to contribute to ALS risk in the brain (Additional file 1: Table S14). *GPX3* was a brain eQTL which was decreased with the risk allele in both brain SMR meta-analyses (opposite direction to blood) *B*_effects_ = −0.40 and −0.18, *p*-values = 2.7 × 10^−23^ and 1.4 × 10^−2^ (PsychENCODE and brain-meta, respectively). Both of the top SNPs did not pass the HEIDI test to infer the causal SNP (not unexpected since the sample size is still small). One SNP was identical to top blood eQTLgen SNP (rs12519636, Brain-meta) and one that was in moderate LD with the top GWAS SNP (rs4958874, *r*^2^ with rs10463311 = 0.61). *TNIP1* was not replicated in either of the eQTL brain SMR meta-analyses (Additional file 1: Table S14-S15).

### Prioritised genes using TWAS

GWAS summary statistics and different transcriptome-wide association study (TWAS) models were used to detect significantly associated genes. This looked at the proportion of tissue-shared and tissue-specific expression (CONTENT and tissue-by-tissue elastic net) to identify 11 genes that passed a conservatively adjusted *p*-value threshold [44]. *ATXN3*, *C9orf72*, *SCFD1*, *CAAP1*, *M0B3B*, *PLAA*, *SHMT2*, *RP11-114F3.5*, *TRIP11*, *ZSWIM8* and *DYNLL2* all demonstrated at least one genetic component of expression that is associated with ALS (Additional file 1: Table S16-S17). While these genes need further follow-up, none was linked to the locus in chromosome five. To identify tissue-specific expression patterns for *GPX3* and *TNIP1*, we used TWAS-CONTENT. Both genes had a significant heritable component (1.7 × 10^−40^ and 1.8 × 10^−6^, respectively), and profiling the total variability explained by the CONTENT found tissue-specific patterns of expression were each detected in brain tissue (Additional file 2: Fig. S6). The findings do not distinguish *GPX3* or *TNIP1* but do identify tissues to follow up, including particular regions of the brain (the frontal cortex) that are common to both *GPX3* and *TNIP1* (Additional file 1: Table S18).

### Gene expression variability analysis of the top two candidates: *GPX3* and *TNIP1*

Given that the SMR results indicate that the risk allele of rs10463311 is associated with increased expression of *GPX3* and decreased expression of *TNIP1*, we used GTEx consortium data to investigate whether, across individuals and tissues, there was evidence for a relationship between *GPX3* and *TNIP1* expression. *GPX3* had higher levels of expression overall (Additional file 2: Fig. S2) but a correlation between the two could imply a common functional relationship [63]. As a benchmark, we initially tested the correlation between their known ALS partners. *GPX3* and *SOD1* are involved in reactive oxygen species degradation, and their correlation was 0.18 ± 0.07 across all tissues (Additional file 2: Fig. S7A). *TNIP1* and *OPTN* are negative regulators of NF-kappa-B signalling (correlation of 0.36 ± 0.07) and *TNIP1* and *TBK1* interact via ubiquitin-binding domain to restrict inflammatory response (correlation 0.12 ± 0.07) (Additional file 1: Table S19, Additional file 2: Fig. S7A). When we tested *GPX3* and *TNIP1*, we found the average correlation of expression across tissues was positive (significant in > 50% tissues, *p* < 0.0001) and was similar or higher than their respective known ALS partners, 0.28 ± 0.07. This correlation was above the median correlation (0.12 ± 0.03) of 16 genes (implicated with unequivocal evidence in ALS and ALS overlap syndromes [1]) (Additional file 1: Table S20, Additional file 2: Fig. S7B).

### Gene-level Polygenic Priority Score

Next, we calculated the Polygenic Priority Score (PoPS) [23] for each chromosome (Additional file 1: Table S21). This is a similarity-based gene prioritisation method which integrates the full polygenic GWAS signal with gene features (derived from RNA expression data, 73 single-cell datasets, predicted protein-protein interaction networks and pathway data) that were not simultaneously considered in other tools, to rank priority genes. It excludes the locus of interest (i.e. chromosome 5) and then uses marginal feature selection to perform enrichment analysis for each gene feature separately before retaining features that pass a nominal significance threshold (*p* < 0.05), to reduce the noise and computational complexity of fitting the joint model. These features are then used to score on the locus of interest. Of the 848 genes on chromosome 5, *GPX3* was ranked first (score: 2.15) (followed by *MEF2C* and *CD74*, scores: 1.93 and 1.66, respectively) (Additional file 1: Table S21) while *TNIP1* was ranked 522nd (score: −0.055). To determine the sensitivity of this result without the *C9orf72* signal on chromosome 9, we re-ran the analysis by removing this locus. This had a minimal effect on ranks and scores (i.e. *GPX3* = 1st, *TNIP1* = 656th) and may reflect different pathways of action to *C9orf72*.

### GPX3 expression is altered with disease stage in ALS cases

To test the relationship between GPX3 expression and disease, we examined available RNA microarray data in an ALS case-control cohort from the Netherlands (*N*_total_ = 942 blood samples, *N*_cases_ = 347/*N*_controls_ = 595) [47] (Additional file 1: Table S22). There was no difference in either *TNIP1* or *GPX3* expression between ALS cases and controls (*p* = 0.36 and 0.12, respectively), and the lead risk SNP did not identify an association with expression (Additional file 2: Fig. S8A-D). In ALS cases, an additive and recessive model of GPX3 expression was tested with the risk SNP to suggest lower expression in risk allele carriers (*p* = 0.12 and *p* = 0.02, respectively), while for *TNIP1* the *p*-value was n.s. (0.22 and 0.21, respectively).

To examine protein levels of GPX3 and TNIP1 in blood plasma, we ran a sandwich ELISA in a discovery cohort of Australian ALS cases and controls. TNIP1 levels were below limits of detection (*LOD* = 23.5 pg/ml; despite loading the highest concentration possible). Similar to the RNA microarray, the level of GPX3 did not differ between cases and controls and there was no detected association with risk allele genotype (Additional file 2: Fig. S9A-B).

**Fig. 4:**
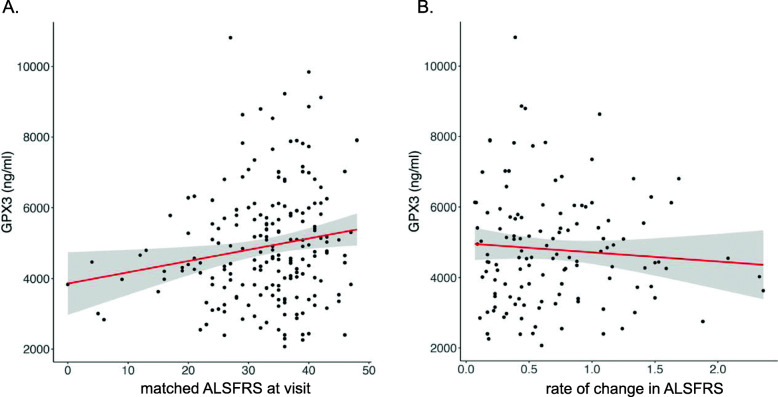
GPX3 protein expression is correlated with clinical measures of ALS function. **A** Linear regression of ALS functional rating score (matched visit) and *GPX3* protein level in plasma (ALSFRS-R, a measure of physical function, high score implies better function) (sex included as a covariate) identifies a positive correlation in ALS cases with functional rating score (ELISA assay, *n* = 200, *p =* 0.0055, adjusted *R*^2^ = 0.042, *y* = 3625.4 + 27.4*x*, *SE* 13.3). **B** Linear regression of change in ALSFRS-R per month since time of onset (0 indicates slow progression, 2 indicates fast progression) and GPX3 expression (*n* = 126, *p* = 0.025, adjusted *R*^2^ = 0.042, *y* = 4326.7 − 126.3*x*, *SE* 284.0) (sex included as a covariate). A similar result was identified in a larger cohort when using proxy dates (when onset date was missing) (Additional file 2: Supplementary Methods) (*R*^2^ = 0.031, *p* = 0.049, for every unit increase rate of progression resulted in a −96 ± 137 ng/ml of GPX3 (mean ± SE) (*n* = 190) vs. a decrease of −126 ± 284 ng/ml of GPX3 (mean ± SE))

Interestingly, within cases, we identified a linear association with GPX3 expression and ALS functional rating score (ALSFRS-R (scale from 48 to 0, where 48 is a normal physical function)), *p* = 6.2 × 10^−3^, *R*^2^ = 0.16, *B*_effect_ ± standard error = 125 ± 39 ng/ml/ALSFRS unit, *n* = 50 ALS cases (Additional file 2: Fig. S9C), to suggest a higher ALSFRS correlated with higher GPX3 (sex included as a covariate). The direction was consistent with time since onset (Additional file 2: Fig. S9D). To validate these findings, we used an independent replication cohort of Australian samples (*N* = 200). We found male cases had higher GPX3 protein levels than female cases (*B*_effect_ = 626 ± 249 ng/ml, *p* = 0.013) and thus sex was included as a covariate (Additional file 2: Fig. S10A). Other variables, such as age at onset, *C9orf72* status, bulbar onset and cognitive problems, had no association with GPX3. The change in the level of GPX3 with advancing disease symptoms (Fig. 4A) was consistent with the rate of disease progression (change in ALSFRS since onset (*p* = 2.5 × 10^−2^, adjusted *R*^2^ = 0.042 (Fig. 4B)), days since symptom onset (*p* = 2.5 × 10^−2^, adjusted *R*^2^ = 0.040, Additional file 2: Fig. S10C), King’s Staging Scale (a burden of disease measure) (*p* = 1.1 × 10^−2^, *R*^2^ = 0.040, Additional file 2: Fig. S10D)). Introducing the Gold Coast diagnostic criteria meant *n* = 10 cases did not meet the diagnostic criteria. Reanalysing the data without these samples (*n* = 188) still identified a significant association between GPX3 and ALSFRS (*p*-value = 1.0 × 10^−2^, *R*^2^ = 0.045, adjusted for sex). In all of these analyses, the effect size of the clinical variables and GPX3 was small, relative to the sex effect on GPX3, and thus sex could be driving these associations.

We looked at preliminary data for longitudinal changes for cases that had two or more visits (*n* = 89 individuals, *n* = 224 observations, 1–5 visits) using a linear mixed-model analysis, fitting individual as a random effect. Examining the change in ALSFRS-R in months since first visit and months since diagnosis, we identified a linear decrease of 0.54 points (0. 42–0.66 95% *CI*, *p* = 7.2 × 10^−17^) and 0.10 (0.034–0.17 95% *CI*, *p* = 2.8 × 10^−16^) each month, respectively. The rate of GPX3 change over time was not significant with the mean change per month since the first visit: −7.4 ng/ml (−53.5 to 38.6 95% *CI*) and since diagnosis: −2.18 ng/ml (−17.9–13.50 95% *CI*). We asked whether the GPX3 level could help explain ALSFRS by including both GPX3 and time in the model but there was no association (Additional file 2: Fig. S11).

### No change in DNA methylation in blood between ALS cases and controls

Given the alterations of GPX3 expression in ALS cases and the SMR results for GPX3 and TNIP1, we queried the summary statistics from a methylome-wide association study [46] and conducted a subset of the Australian ALS case-control cohort (*N*_cases_ = 782, *N*_controls_ = 613). We narrowed our query to probes annotated to *GPX3* and *TNIP1* [49]. None of the queried probes was statistically significant in the MOMENT analysis or the beta methylation value comparisons (probes nearest or within these genes) (Additional file 2: Fig. S12).

### In vitro knockdown of *GPX3* or *TNIP1* in differentiated human spinal motor neurons

To understand the functional implications of expression changes in *GPX3* and *TNIP1*, differentiated human spinal motor neurons were used as an in vitro model. Target *GPX3* and *TNIP1* small interfering RNAs (siRNA) constructs were able to knockdown (KD) mean expression by 75.5 ± 8.8% and 53.3 ± 17.3%, respectively, compared to the scrambled siRNA (*p* = 1 × 10^−3^ and *p =* 0.03) (quantification by real-time qPCR). Cell viability assessed using an assay for metabolic activity (MTT reduction assay) showed no significant change following knockdown of either *GPX3* or *TNIP1*. Live-cell imaging conducted on HB9-GFP-labelled motor neurons identified no gross morphological defects and no significant effect on the dominant neurite length, total neurite length, or the number of branches/neurites per neuron (unpaired *t*-test) following *GPX3* or *TNIP1* knockdown (Additional file 2: Fig. S13).

### Knockdown of *gpx3* (but not *tnip1*) in zebrafish-MO is associated with deficits in motor function

**Fig. 5:**
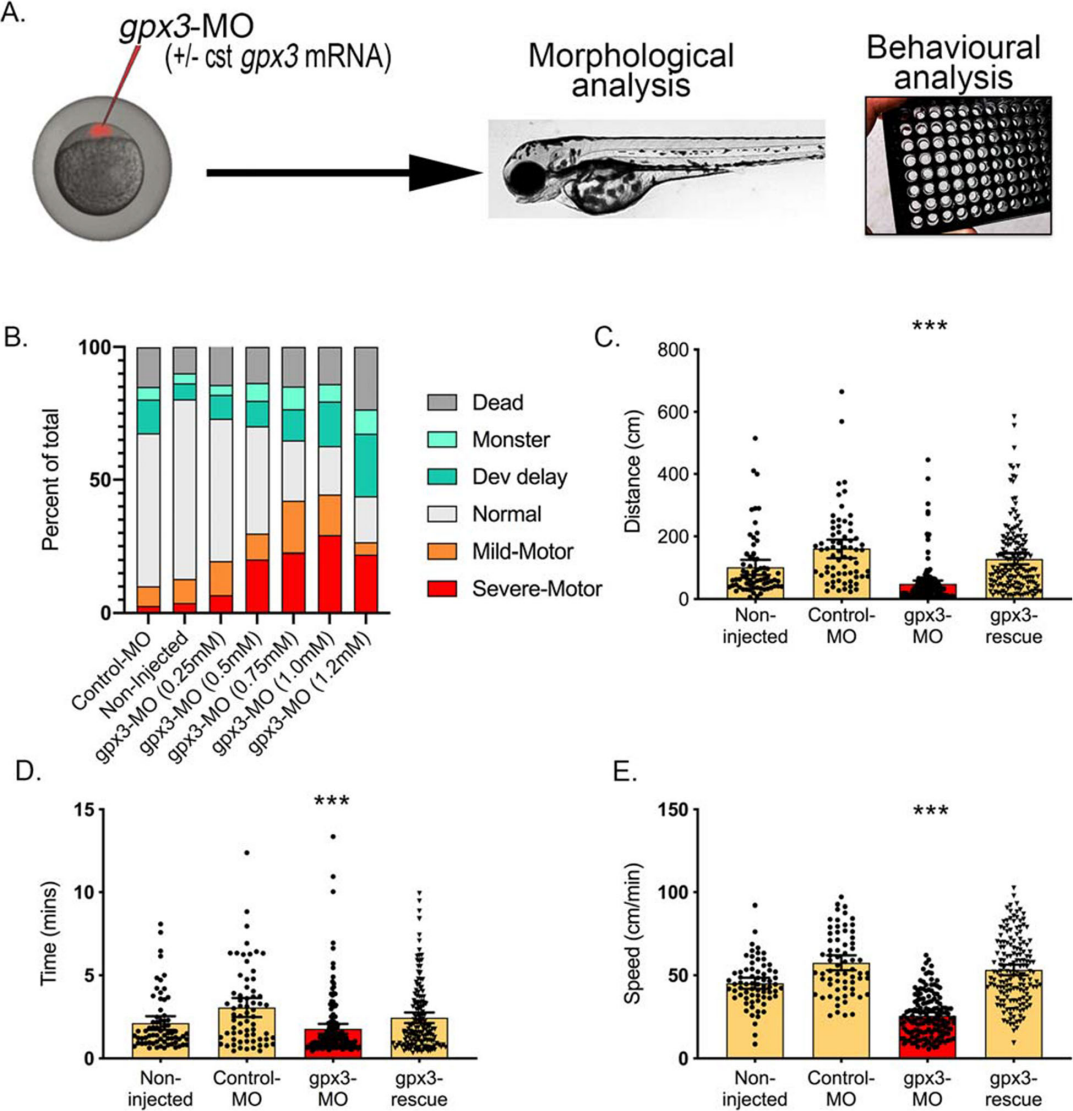
Knockdown of *gpx3* in zebrafish-MO results in a motor phenotype. **A** Zebrafish embryos were injected at the one-cell stage with or without control MO (Control-MO), anti-gpx3 MO (*gpx3*-MO) or combined anti-*gpx3* MO with 100 pg of cst-*gpx3* mRNA (*gpx3*-rescue). Morphological and behavioural analyses were carried out post-fertilisation. **B** A range of MO concentrations targeting *gpx3* indicated a dose-dependent motor-phenotype effect, which peaked at 1 mM without developmental abnormalities (age: 2dpf). **C**–**E** The *gpx3*-MO (1 mM) injected animals moved a smaller distance, for fewer minutes and with an overall lower speed compared to both un-injected and CTR-MO-injected controls. The motor defects were all significantly rescued following co-injection with MO-insensitive *gpx3-*mRNA (cst-gpx3) (*n* = 67–158, mean ± 95% CI). Significance indicates the comparison between control-MO and *gpx3*-MO (*p* < 0.001). Graphs show individual values, mean and 95% CI error bars. Swim distance, time and speed mean difference and 95% CI: 112 ± 28 mm, 1.29 ± 0.59 s and 32.0 ± 2.53 mm/s, respectively

In parallel, we conducted in vivo functional loss-of-function (LOF) experiments (Fig. 5). The zebrafish genome carries 1 orthologue for *GPX3* (*gpx3*) and 1 orthologue for *TNIP1* (*tnip1*). Both are highly conserved, having 98% and 97% amino-acid similarity to the human genes, respectively. Using morpholino-mediated (MO) knockdown of *tnip1* in zebrafish embryos, we did not detect any significant motor neuron development or motor function phenotype from 1 to 5dpf (days after birth) (data not shown). However, injection of anti-*gpx3* MO did impact the motor functions of the zebrafish larvae, without triggering any obvious significant developmental abnormalities such as growth malformation or premature death (Fig. 5B, Additional file 1: Table S23).

Using an optimised dose of *gpx3*-mRNA MO (Fig. 5B), we further investigated the excess number of motor phenotypes by measuring swim distance, time and speed (*n* = 151) compared to mismatch injected controls (*n* = 67). Swim distance, time and speed were significantly shorter in the gpx3-MO compared to the control (mean difference and 95% *CI*: 112 ± 28 mm, 1.29 ± 0.59 s, 32.0 ± 2.53 mm/s, respectively, *p*-value for all < 0.0001) (Fig. 5C–E).

To further validate the specificity of these findings, we co-injected a custom-made MO-insensitive *gpx3*-mRNA (cst*GPX3*) (*n* = 158) and performed the same assay. The presence of the custom gpx3-mRNA significantly reduced (rescued) the observed *GPX3*-MO-induced motor deficits (Fig. 5C–E).

To test the potential pathogenicity/motor phenotype of *GPX3* overexpression, we injected increasing doses of cst*GPX3*. There was no impact to the motor functions of the zebrafish larvae or obvious significant developmental abnormalities across three doses (Additional file 2: Fig. S14, Additional file 1: Table S24). At the dose with (< 50%) death/monster phenotypes (to indicate no significant generalised toxicity) (100 pg (1 mM)), there were < 5% of MO with a mild-motor phenotype (*n* = 5/107) and none with a severe motor phenotype.

## Discussion

Here, we use a comprehensive suite of tools to identify the most likely target gene for ALS risk on chromosome five. It follows up our initial trans-ethnic GWAS findings [4] and utilises in silico, in vitro and in vivo approaches (Fig. 1) to prioritise *GPX3* and lend some support for *TNIP1*. While we focus on chromosome five, candidate genes detected in other loci could be further investigated using relevant in vivo and in vitro models. Our approach is atypical with few previous examples of similarly in-depth investigations of risk loci in ALS (and other complex neurological/neurodegenerative diseases) and thus our multi-platform approach (Fig. 1) with other exemplars [15–17] could be applied to new GWAS-risk loci in the future.

ALS is a complex, polygenic disease, and our initial results using the latest ALS GWAS summary statistics [9] provided basic reassurance that the chromosome 5 locus could be a true positive despite concern that the SNP-based heritability estimated from these data was low (*h*^2^_SNP_ = 0.018 ± 0.0038). Integration of GWAS results with independent gene expression data showed results that are consistent with the neurodegenerative disease processes in ALS [59, 60]. Functional annotation enrichment was also consistent with other GWAS results [21] and included enrichment in intronic regions, areas of active methylation and open chromatin. Prioritising 92 genes by functional mapping [19], we identified enrichment of expression in the brain (GTEx), neuron-specific top gene ontology (GO) pathways and a heritability enrichment of cell types that included the central nervous system, frontal lobe, dendritic cells and muscle (FUMA, S-LDSC).

Using conditional and joint approach (GCTA-COJO) [34] identified one signal driving the significant GWAS association, and the SNP with the strongest support was rs10463311. Positional mapping, expression quantitative trait loci (eQTL) (GTEx) [28] and chromatin interaction mapping indicated locus links to five genes using FUMA [19] but only two, *GPX3* and *TNIP1*, were consistently linked across all three techniques (Fig. 2). Independently, the use of the gene prioritisation tool, PoPS [23]_,_ to combine gene features based on expression, pathways and protein interactions ranked *GPX3* as the lead candidate (top in chromosome 5) with little support for *TNIP1* or other candidates. PoPS has good sensitivity for prioritising known genes for other conditions but is yet to be tested for ALS, and as recommended [23], follow-up analyses are still required.

To test if the SNP-ALS association could be mediated through a SNP-gene expression association, we ran SMR using the largest available eQTL sample for whole blood (*n* = 31,684) to identify *GPX3* and *TNIP1*. The top eQTL SNP (rs1258386, *p*_eQTL_ = 1.1 × 10^−171^) was in low LD (*r*^2^ = 0.13) with the top GWAS SNP (rs10463311). Conditioning on the top GWAS SNP (rs10463311) (Additional file 1: Table S10) is suggestive that the rs12518386 GWAS association is not driven by shared LD alone (ALS association *p*-value for rs12518386 retained a significant effect after conditioning on rs10463311, *p* = 2.1 × 10^−3^, *B*_effect_ = 0.049 out of 0.078) and thus a second locus may be identified in future GWAS studies. Consistent with this, the associations did not pass the HEIDI test which meant a simple relationship between the associated SNP and ALS via gene expression could not be determined. Despite no causal SNP identified (given the complexity of the region), the GWAS-associated risk allele and its correlated rs1258386 eQTL allele increased *GPX3* expression and decreased *TNIP1* expression. The difference in direction of expression in blood is notable given that across tissues the mean levels of expression between *GPX3* and *TNIP1* are positively correlated (0.28 ± 0.07, Additional file 2: Fig. S7).

Given the brain is a relevant tissue in ALS (but remains much smaller in size to blood expression data), we tested if the six identified significant blood eQTL genes were also detected in the brain using a nominal threshold. Four genes discovered in blood tissue were also significant in brain eQTL SMR analysis, to indicate that these genes (*GPX3*, *C9orf72*, *SCFD1*, *RP11-529H20.6*) are also likely to contribute to ALS risk in the brain (Additional file 1: Table S14). Interestingly, the brain eQTL SMR results identified that chromosome 5 risk locus decreased *GPX3* expression which was opposite to blood (Additional file 1: Table S14). While further investigations are needed to understand why the risk allele may differentially alter the expression of *GPX3* in blood vs. brain (a phenomenon in ~ 5% of blood cis-eQTLs [64]), also pertinent to examine was the level of expression in an ALS cohort.

Examining expression in those diagnosed with ALS found that the chromosome 5 risk locus correlated with a lower GPX3 level (TNIP1 level could not be detected). Two independent ALS cross-sectional studies, while being underpowered (Additional file 2: Fig. S15, [65]), suggested risk allele homozygotes had lower levels of GPX3 expression (*p* = 0.02 and *p* = 0.06, microarray and protein, respectively). This change in expression with the risk allele was consistent with brain eQTL data. Pairing GPX3 expression levels with ALSFRS score (a clinical questionnaire assessing functional disability and extent of neuronal loss) demonstrated that GPX3 expression was lower in those with a more progressed disease (*p* = 6 × 10^−3^, *B*_effect_ = 125 ± 39 ng/ml/ALSFRS unit, *n* = 48 ALS cases, adjusted for sex). This was replicated in a larger, independent cohort (*p =* 5.5 × 10^−3^, *n* = 198), and while the correlation was again relatively weak (*R*^2^ = 0.042, *B*_effect_ = 27.35 ± 13.3 ng/ml/ALSFRS unit, adjusted for sex), it suggested a reduction in GPX3 corresponded to lower in ALSFRS. This result was similar when looking at the rate of disease progression, those with a lower GPX3 level had a faster rate of progression (*R*^2^ = 0.042, *p* = 2.5 × 10^−2^) (Fig. 4B). These findings are reminiscent of previous findings in a *SOD1* rat model of ALS [14] that identified significantly lower levels of gpx3 at the end stage of disease, compared to wild-type age-matched controls [14]. Interestingly, there was a higher gpx3 level in *SOD1* pre-symptomatic rats (when the highest levels of oxidative damage were identified), to suggest *GPX3* levels are dynamic in blood both prior and during disease.

We did carry out a preliminary longitudinal analysis of ALS cases, but this did not reveal an association with time (despite a significant relationship between ALSFRS and time). It is relevant to acknowledge that prevalent ALS participants (i.e. those well enough to attend multiple clinics over a long period) can inevitably bias time-based analyses and none was enrolled prior to symptom onset.

*TNIP1* expression analyses were more difficult to assess as protein levels were below the limits of ELISA detection and, on microarray, there were no differences between ALS cases and controls.

To rapidly characterise the functional impact of these prioritised genes, we used in vivo (zebrafish embryo) and in vitro (human motor neurons) models amenable to expression changes. Manipulation of zebrafish embryos fitted these requirements and perturbation of expression has previously been used to identify genes contributing to neurodegenerative diseases including ALS [66, 67]. The *tnip1* knockdown did not trigger any obvious motor phenotype in the zebrafish-MO, consistent with two other reported *tnip1*-MO knockdown results [68, 69]. However, the *gpx3-*loss-of-function (LOF/knockdown) zebrafish larvae did have a phenotype, with decreased motor functions, with swim distance, swim time and overall speed significantly reduced without any obvious morphological defects. Importantly, the observed motor phenotypes were rescued via co-expression of a synthetic *gpx3* mRNA (insensitive to the injected anti-*gpx3* MO). In contrast, *gpx3* overexpression did not show any specificity for a motor phenotype. This is consistent with in vitro [70, 71] and in vivo [72] reports that overexpression of *GPX3* has been shown to protect, rather than damage cells. While the suite of gpx3-MO support loss of GPX3 affecting motor function (rather than gain), further studies would be required to further understand the pathogenic role of *gpx3* on the motor function in our zebrafish model.

Modelling knockdown in vitro in human motor neurons using an siRNA approach, we found no gross alterations in motor neuron development or survival for either gene. This result was expected given that genes associated with ALS risk and even pathogenic ALS mutations may have only subtle effects and this model may not be sensitive enough to detect these. Other specific assays testing electrophysiology properties [73], age acceleration [74] or other cell types involved in neuronal health may be more relevant (but are also yet to be tested for sensitivity to risk loci). Further characterisation is needed with particular attention made to address these limiting factors. No known ALS risk genes were identified in a recent CRISPRi screen [75] (day 7 neurons), while a known causal ALS gene, *SOD1*, was found to alter survival (day 14 and day 28). Interestingly, when neurons were stressed with the knockdown of essential survival genes (UBA1/MAT2A) both *TNIP1* and *GPX3* expression significantly increased in single-cell RNA sequencing. Notably, their expression pattern changed alongside many thousands of other genes and thus it remains important to elucidate their contribution to neuronal function both in vitro and in vivo, particularly as non-cell autonomous mechanisms (disease arising from a combination of motor neurons and their cellular partners) have been proposed in ALS [1].

**Fig. 6:**
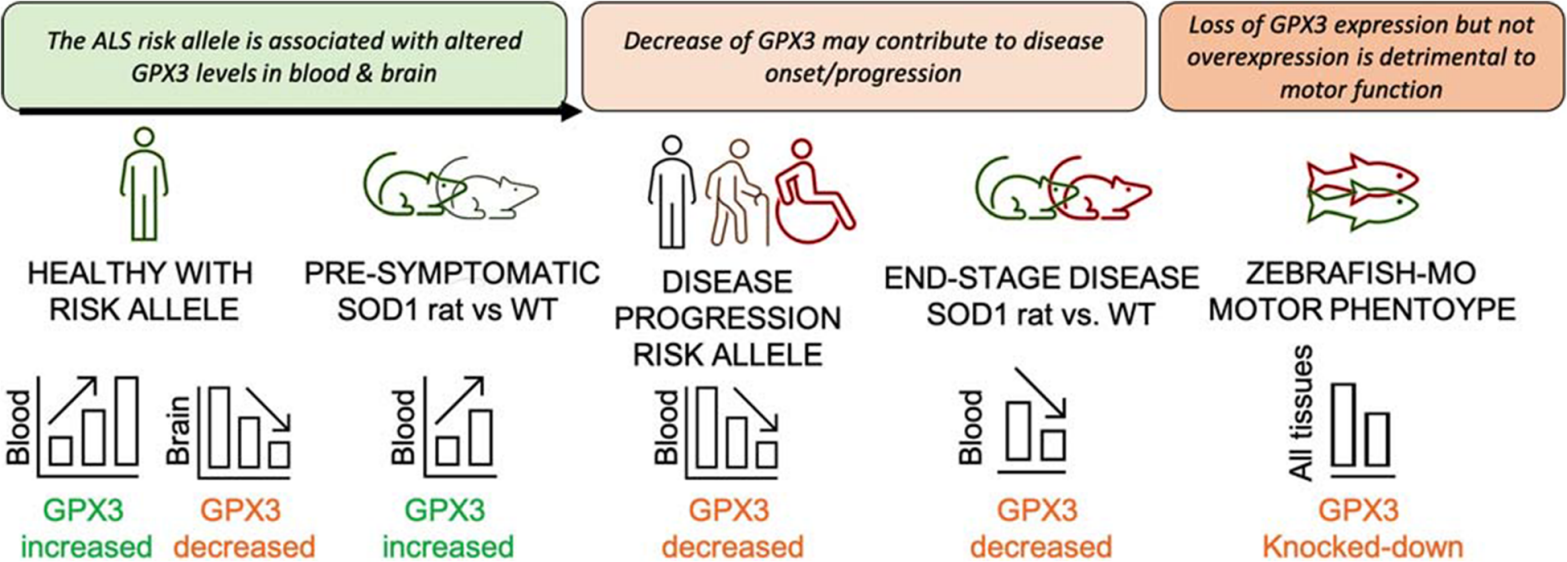
Proposed mechanism to explain GPX3 expression with respect to genotype and disease. In healthy individuals carrying an ALS risk allele, GPX3 levels are increased in blood but decreased in the brain compared to non-carriers (SMR from ALS GWAS, eQTLgen *n* = 31,684 and brain meta-analyses *n* = 2581). The ALS SOD1^H46R^ rat model [14] shows an increased level of GPX3 compared to wild-type (WT) controls prior to disease onset. This might indicate GPX3 is being secreted into the circulation to support homeostasis/healthy cellular function via antioxidant mechanisms. In disease, cross-sectional analysis of ALS cases, there is reduced expression level with a more progressed disease (Fig. 4), and at end-stage of disease in SOD1^H46R^ rats, GPX3 levels are lower vs. WT controls. Analysis of cross-sectional ALS cases data shows a trend for homozygotes of the risk allele to have lower levels of GPX3 (microarray and protein, *p* = 0.02 and *p* = 0.06 respectively, Additional file 2: Figs. S8 and S9). Independently, an in vivo zebrafish-MO model with knockdown of endogenous *gpx3* demonstrates a motor phenotype (reduced swimming time, distance, speed) that is rescued with *gpx3* expression, to support its essential role in motor function (Fig. 5), while overexpression of variable doses of *GPX3* does not result in a motor specific phenotype (Additional file 2: Fig. S14)

Our *GPX3* analysis suggests that the protein expression of GPX3 decreases with disease progression and its levels may relate to the ALS GWAS-risk locus (Fig. 6). The motor phenotype of the zebrafish-gpx3-MO model supports *GPX3* as a potential disease modifier and therapeutic target in ALS. Oxidative stress is one of several mechanisms directly linked to ALS via causal gene mutations [1] and resonates with the known functions of GPX3. As a glutathione peroxidase, it is part of the body’s arsenal of antioxidant enzymes, reducing oxidative stress (downstream of superoxide dismutases (SODs)) by scavenging hydrogen peroxide in the presence of reduced glutathione [76]. *GPX3* is a distinct glutathione peroxidase as it is secreted (produced in the kidney) and found abundantly in blood plasma and other tissues, including neurons and the brain (Additional file 2: Fig. S16) [75, 77]. Deficiency of *GPX3* in humans [78, 79] has been associated with stroke and ischemic heart disease, and *Gpx3-*knock-out mice have a prothrombotic state and vascular dysfunction due to the accumulation of reactive oxygen species (ROS) [80]. The presence of oxidative stress biomarkers in ALS and animal models of *SOD1* mutations support a crucial role for cellular antioxidant defences in stopping cell death [81, 82]. Interestingly, increased levels of *SOD1* and *GPX* have been suggested to protect neuronal cells from antioxidant damage [83, 84] and maybe a relevant therapeutic to investigate for those with ALS. GPX3 is a selenoprotein as it contains a selenocysteine (Sec) codon (UGA) and thus transcription can be altered by dietary/exogenous selenium. Interestingly, the use of Ebselen, an organo-selenium compound that increases gpx3 and reduces mutant SOD1 aggregation [85], was considered neuroprotective as it delayed disease onset in SOD1^G93A^ mouse (but not survival) [86]. The establishment of stable zebrafish lines to mechanistically address how the level of GPX3 might contribute and/or abate oxidative stress and how this may be a contributing cause and/or effect of ALS is an avenue for further follow-up.

Associations with *TNIP1* were more difficult to detect in human ALS cohorts, and no changes in vivo were identified. Additional analyses are needed to determine if *TNIP1* is also a genuine target with the possibility that both genes in this single locus contribute to disease risk.

Further investigations into the mechanisms are warranted, and we note several limitations to our study. The correlations we have performed with protein levels of GPX3 and ALSFRS and progression, while replicated, do not specifically indicate the cause or risk associated with disease and further in-depth analyses are needed. One such analysis for follow-up is the effect of sex and *GPX3* expression. Our replication cohort detected higher GPX3 protein expression in male cases. The GPX3/sex effect was larger than the subsequently tested clinical associations such as rate of progression and time since onset and thus could be driving these associations. Larger sample sizes will help to determine the role of sex, disease progression and other clinical variables (i.e. onset location) and levels of GPX3. Carrying out these expression analyses (overexpression and knock-out) in a variety of cell types, tissues or models, including the brain motor cortex/motor neurons, expected to be more salient for risk than those that are currently available may be helpful in this regard. Specific to our GWAS results, with our current sample size and available data, we cannot rule out causality/pleiotropy from linkage at this locus. The ALS GWAS sample sizes are expected to increase which will generate more associated regions, and in the future, there will be improved high-throughput methods to follow up each associated locus in ALS for in-depth investigations in a consistent approach. Examining sequencing data in the future may be useful as current data reveals very few loss of function *GPX3* variants [87]. These are rare (< .0001) and none is in a homozygous state and so it cannot be determined whether or not they contribute to ALS. Future SNP-array/sequencing studies across different ancestries remain relevant, particularly as the lead GWAS risk allele (rs10463311) is more frequent in East Asian (0.48) vs. European (0.26) ancestry populations. We highlight that investigation of common-loci contribution in neurological conditions is not well determined and future development of efficient model systems that are sensitive to detect risk genes rather than causal genes remains important and relevant for the research community. Despite these limitations, we do not expect future analyses to contradict the bulk of our results, all of which complement the genomic studies of this disease (and previous literature) and could be used as a basis for future investigations.

## Conclusions

To conclude, we report *GPX3* as a lead target to investigate in ALS risk with support from human-derived data and a motor phenotype in a zebrafish model. While additional characterisation is still needed, i.e. larger studies may help elucidate the link between the causal SNP and *GPX3*, and/or in vivo models may investigate modulating its expression, our findings support it as a lead candidate relevant to understanding mechanisms of disease and therapy development in ALS. We note that for follow-up analyses it was difficult to rule out the contribution of *TNIP1* at this locus. *TNIP1* was implicated in silico and in SMR but not in disease cohorts or with the in vivo zebrafish model. Future studies should still consider whether *TNIP1* has a role in ALS risk and whether this is independent of *GPX3* (given their correlated expression). With few ALS treatments available, and the majority of those with ALS not identified with a single causal mutation [88, 89], starting pre-clinical studies based on candidates derived from human ALS GWAS follow-up studies could be a worthwhile avenue to pursue.

## Supplementary Information

**Additional file 1: Supplementary Tables. Table S1**: Significant risk loci detected from ALS case-control GWAS. **Table S2**: Lead SNPs identified from independent significant SNPs of ALS GWAS. **Table S3**: Independent significant SNPs at r2 < 0:6 identified from ALS GWAS. **Table S4**: FUMA identifies 92 genes (ALS GWAS). **Table S5**: Prioritized genes from ALS GWAS by functional mapping. **Table S6**: FUMA annotation pathway categories. **Table S7**: S-LDSC annotation categories enriched in ALS GWAS. **Table S8**: S-LDSC cell-type categories enriched in ALS GWAS. **Table S9**: GCTA-COJO analyses. **Table S10**: GCTA-COJO analysis conditioned based on lead SNP (rs10463311) and different co-linearity thresholds. **Table S11**: FUMA chromatin interactions. **Table S12**: SMR results for GPX3, TNIP1 and C9orf72. **Table S13**: SMR significant genes. **Table S14**: Brain eQTL SMR results to follow-up significant genes in blood eQTL data. **Table S15**: The top GWAS and SMR chromosome 5 SNPs in brain expression datasets. **Table S16**: Significantly associated genes using TWAS models elastic net and CONTENT models. **Table S17**: Significantly associated genes using TWAS models elastic net and CONTENT models for each identified tissue. **Table S18**: TWAS CONTENT full model with significantly expressed GPX3 or TNIP1 in relevant tissue types. **Table S19**: Gene correlation in 48 GTEx tissues. **Table S20**: Gene correlation of 16 ALS genes in 45 GTEx tissues. **Table S21**: Top two prioritized candidates per chromosome in ALS GWAS using PoPS. **Table S22**: Platforms used for microarray ALS and control samples and genotype counts. **Table S23**: Morpholino (MO) abnormalities after injection of GPX3-knock-down mRNA were no different from controls or non-injected MO. **Table S24**: No severe motor abnormalities in zebrafish morpholinos (MO) with increasing GPX3 mRNA.

**Additional file 2: Supplementary Methods and Figures. Fig. S1**. Functional Annotation and Mapping (FUMA) histogram summary. **Fig. S2**. Functional Annotation and Mapping (FUMA) gene expression heat map. **Fig. S3**. S-LDSC annotation enrichment in ALS GWAS. **Fig. S4**. S-LDSC finds CNS and musculoskeletal cell-type categories are enriched in ALS GWAS. **Fig. S5**. Summary statistics-based Mendelian Randomisation (SMR) analysis identifies *GPX3* and *TNIP1.* **Fig. S6**. TWAS-CONTENT full model GPX3 and TNIP1 gene expression variance. **Fig. S7**. Gene correlation across tissues. **Fig. S8**. Microarray expression data of TNIP1 and GPX3 demonstrate no difference in expression between cases and controls or genotype. **Fig. S9**. Preliminary Discovery cohort data demonstrated association with GPX3 and ALS. **Fig. S10**. Replication GPX3 cohort. **Fig. S11**. Preliminary longitudinal data assessment of ALSFRS-R and GPX3 levels. **Fig. S12**. *TNIP1* and *GPX3* methylation in ALS and control blood. **Fig. S13**. Knockdown of *GPX3* and *TNIP1* in human motor neurons. **Fig. S14**. Motor and developmental effect of *GPX3* overexpression in zebrafish. **Fig. S15**. Effect-size sensitivity analysis. **Fig. S16**. GPX and GPX3 expression pattern across tissues. **Fig. S17**. Gpx3 amplicon for danio rerio. **Fig. S18**. pME-dre_gpx3_Codon_optimised plasmid.

## Data Availability

The plasma expression data and full results for FUMA, PoPS, TWAS and zebrafish-MO tracking data are hosted at https://github.com/CNSGenomics/ALS-GPX3-TNIP1 [90]. The FUMA job results are also published online (SNP2GENE jobID*366) https://fuma.ctglab.nl/browse.
