# Supplementary material for: Functional characterisation of the amyotrophic lateral sclerosis risk locus GPX3/TNIP1
Source: Genome Med. 2022 Jan 19;14:7. doi: 10.1186/s13073-021-01006-6 (PMC8767698; doi:10.1186/s13073-021-01006-6)
Supplement: Supplementary file 2 — Additional file 2: Supplementary Methods and Figures. Fig. S1. Functional Annotation and Mapping (FUMA) histogram summary. Fig. S2. Functional Annotation and Mapping (FUMA) gene expression heat map. Fig. S3. S-LDSC annotation enrichment in ALS GWAS. Fig. S4. S-LDSC finds CNS and musculoskeletal cell-type categories are enriched in ALS GWAS. Fig. S5. Summary statistics-based Mendelian Randomisation (SMR) analysis identifies GPX3 and TNIP1. Fig. S6. TWAS-CONTENT full model GPX3 and TNIP1 gene expression variance. Fig. S7. Gene correlation across tissues. Fig. S8. Microarray expression data of TNIP1 and GPX3 demonstrate no difference in expression between cases and controls or genotype. Fig. S9. Preliminary Discovery cohort data demonstrated association with GPX3 and ALS. Fig. S10. Replication GPX3 cohort. Fig. S11. Preliminary longitudinal data assessment of ALSFRS-R and GPX3 levels. Fig. S12. TNIP1 and GPX3 methylation in ALS and control blood. Fig. S13. Knockdown of GPX3 and TNIP1 in human motor neurons. Fig. S14. Motor and developmental effect of GPX3 overexpression in zebrafish. Fig. S15. Effect-size sensitivity analysis. Fig. S16. GPX and GPX3 expression pattern across tissues. Fig. S17. Gpx3 amplicon for danio rerio. Fig. S18. pME-dre_gpx3_Codon_optimised plasmid. [file 13073_2021_1006_MOESM2_ESM.docx]

**Additional file 2: Supplementary Methods and Figures**

**Functional characterisation of the Amyotrophic Lateral Sclerosis risk locus *GPX3/TNIP1***

Restuadi et al.

**Supplementary Methods**

**FUMA**

The 11 single-cell data sets were selected based on LDSC-R cell-type enrichment results. This consisted of human brain (PsychENCODE, Allen_Human_LGN_level1, Allen_Human_MTG_level1, GSE104276_Human_Prefrontal_cortex_all_ages, GSE67835_Human_Cortex_woFetal, Linnarsson_GSE101601- Human_Temporal_Cortex and Midbrain), human blood (human) (GSE89232_Human_Blood, PBMC_10x_68k) and muscle (Mouse_Cell_Atlas_muscle, TabulaMuris_FACS_Limb_Muscle).

**TWAS-CONTENT detail**

We provide additional detail the assumed generative model and objectives of CONTENT (41). It is based on the methodology and decomposition of a previous work by Lu et al., FastGxC (42). Firstly, the method decomposes the genomic measurement of each individual across contexts into between-individuals and within-individuals components. Next, it estimates the contribution of genetic variability on the tissue-shared component. The method then treats the tissue-specific variability as a deviance from the tissue-specific component to remove the portion of the noise that is correlated across tissues. In this way, the method assumes that in the given expression of a gene, there is a genetic component of expression that is shared across all tissues, and a genetic component of expression that is tissue-specific.

We assume that the expression of an individual in a given gene and tissue is a combination of a tissue-shared genetic component that is shared across different tissues and a tissue-specific genetic component that is specific to a tissue, that is;


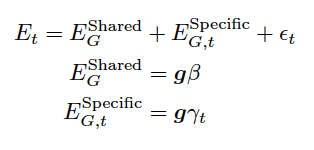


where Et denotes the expression of the individual at the gene in tissue t, EGShared and EGTSpecific denote the components of the expression due to shared and specific tissue genetic effects respectively, *B* and t represent the shared and specific genetic effects respectively, and Et ~N(0; delta 2 t) represents the environmental effects on the individual's gene expression.

The objective of CONTENT is to build a genetic predictor of context-specific phenotypes. While previous work has focused on building powerful genetic models for E, we aim to build unbiased models that partition and estimate the homogeneous g*B* and heterogeneous terms gYt. Specifically, we aim to maximize the power to detect the heterogeneous terms, allowing some leniency in the accuracy of homogeneous terms, as we are interested in context-specific effects. Moreover, as a tissue-specific predictor can be used in downstream analyses to identify the specific context(s) through which genetic variation manifests its effect on the phenotype and disease risk, we also aim to minimize the correlation between the predicted tissue-specific component and the true tissue-shared component. Finally, our method must account for the shared noise of individuals across contexts and do so in a computationally efficient manner.

Many genomic datasets, such as those of GTEx, have a multilevel nature; with first, individuals sampled and second that same individual is sampled in different tissues contexts. To learn which tissues contribute to a phenotype, we focus our analysis on the GTEx dataset. To take the multilevel structure of the data into account, the observed expression on gene j can be decomposed into an offset term, a between-individual component and a within-individual component. That is, if E_ijti_ denotes the observed expression levels for individual i (i = 1…. I) on gene j (j = 1…. J) and tissue ti (ti = 1….. Ti), E_ijti_ can be decomposed as:


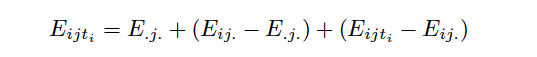


where E.j. = 1TPIi=1PTiti=1 Eijti the mean expression of gene j computed over all (I) individuals and all tissues measured for each individual (T = PI i=1 Ki), and Eij: = 1 TiPTit=1 Eijti the mean expression of individual i on gene j, computed over all tissues of individual i (Ti).

In (1), E.j. is a term that is constant across individuals and tissues for each gene, (Eij. – E.j. ) is the between-individuals deviation, and (Eijti – Eij. ) is the within-individuals deviation of the expression on gene j .Variables that differ between but not within individuals, e.g. sex and genotype, will have an effect on (Eij. – E.j.) but not on (Eijti – Eij. ). On the other hand, variables that change within individuals but are the same between individuals, e.g. the genetic effect on a specific tissue, will have an effect on Eijti – Eij.) but not on (Eij. – E.j.).

In the context of estimation, we center and scale the expression of gene (j) in each tissue (ti) to be:


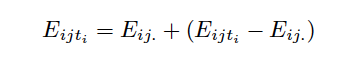


The simplified decomposition equation above is then used to build genetic predictors of context-specific effects while accounting for shared noise. Intuitively, the between-individuals variability serves as the component of expression that is shared across tissues, and the deviance from this shared component (i.e. the within-individual variability) serves as the tissue-specific component of expression. Moreover, treating the tissue-specific component as a deviance from the tissue-shared component leads the decomposition to have the property that as the correlation in residuals between tissues increases, the power to detect tissue-specificity also increases. In addition, the decomposition generates tissue-shared and tissue-specific components of expression that are orthogonal to each other.

For a single gene j, CONTENT takes as input centered, scaled, and residualized (over a set of covariates) expression measured across I individuals in T tissues and an I_m genotype matrix Gj with m measured cis-SNPs for gene j. CONTENT then decomposes the expression vectors into T tissue-specific components and a single tissue-shared component by simply calculating the mean of expression for each individual across their available Ti tissues, and setting the tissue-specific expression for tissue ti as the difference between the observed expression of tissue ti and the calculated tissue-shared expression. As it has been observed that cis-genetic e_ects may be sparse and that the elastic net may perform best relative to other penalized linear models in the context of genetically regulated gene-expression (41). CONTENT fits T + 1 penalized linear models for the T + 1 expression components using an elastic net. Lastly, CONTENT generates a final genetic predictor of expression by combining the tissue-shared and tissue-specific components. Importantly, as the tissue-specific component is a deviance from the tissue-shared component, the sign of the tissue-specific component must be properly realigned when combining both components of expression to make a final predictor. We refer to this linear combination of expression components as the full model of CONTENT and fit it using a simple linear regression.

1. Obtain EShared and ESpecific from the decomposition.

2. Generate cis-genetic predictors of each component using elastic net:

**
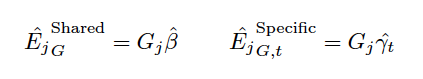
**

3. Regress the expression of tissue, t onto the tissue-shared and tissue-specific components:

**
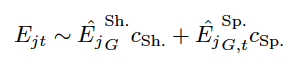
**

For each gene, the set of regression weights is saved, cShared and cSpecific from the last equation for use in downstream analyses. Namely, in TWAS, each tissue receives a single vector of weights, and to test the association of a gene-tissue's full model to a trait, a weighted sum of the predictors is learned from equation (2), cShared β^ + cSpecific γ^t. The same procedure is used for the tissue-specific weight to ensure the correct directionality. To test for significance of genetic effects (i.e. to call an eGene or eAssociation), we correlate each component of expression - the tissue-shared, tissue-specific, and full- to its corresponding genetically-predicted value.

**GPX3 protein expression in blood plasma**

GPX3 activity was detected in plasma samples and after a standard curve optimisation following kit instructions (Adipogen, Liestal Switzerland, cat: AG-45A-0020YEK-KI01). Case and control plasma was diluted at 1 in 200 (ELISA Buffer 1X) and run in duplicate for the assay following kit instructions. Briefly, diluted plasma and standards were added into the wells for binding to the coated antibody plate and incubated at 37 degs for 1 hour. The same standard was used across all plates. After aspirating wells and washing 3 times to remove unbound compounds and the detection antibody was added. Following the second incubation (37°C for 1 hour) wells were again aspirated and washed to remove any excess biotinylated antibody before diluted HRP-labelled Streptavidin was added. To measure the level of the GPX3 in the sample, wells were aspirated and washed before peroxidase activity was quantified using the substrate 3,3’,5,5’-tetramethylbenzidine (TMB). After 20mins at room temperature, stop solution was added and the reaction measured at 450 nm after acidification (Microplate reader, Infinite® M1000, Tecan, Morrisville NC, USA).

**GPX3 expression and rate of disease progression**

Given GPX3 was associated with clinical score of ALSFRS, we tested whether it was also associated with rate of progression. The rate of progression was calculated using the following formula:

Δ ALSFRS = (48 - ALFRS score at visit date) / (months between onset date and visit date)

Onset date was missing for n=72 individuals in the cohort and thus we examined the correlations with two date proxies 1) multi-visit data and 2) diagnosis date to determine if we could use these values if onset date was not available. Briefly, for individuals that had multiple visits, we calculated months between the first and last visit and the change in ALSFRS. For those that had a diagnosis date, we took one month earlier (this was based on the median difference in our cohort between diagnosis and symptom onset (n=10)) and used an ALSFRS of 48. The ‘multi-visit’ rate of disease progression had a stronger correlation (with standard ‘onset date’ calculation, R^2^= 0.91, p< 2.2x10^-16^, n=126) vs. the ‘diagnostic date’ rate of disease progression calculation (R^2^= 0.60, p< 2x10^-16^, n=120). Therefore, we used rate of progression calculated from multi-visit as proxy (n=19) followed by rate of progression calculated from diagnosis date (n=45) as a proxy. This meant rate of progression and GPX3 level could be assessed in n=190 cases.

***In-vitro* knock-down and over expression of *GPX3* and *TNIP1* in Motor Neurons**

Target siRNA knock-down of *GPX3* or *TNIP1* gene expression was confirmed with real-time qPCR using primer sequences below:

| *GPX3* primers | Fwd: ATGCTGGCAAATACGTCCTC  Rev: AGAATGACCAGACCGAATGG |
| --- | --- |
| *TNIP1* primers | Fwd: TGAGCAATGGCAACAAAGAG  Rev: ACCACCTCTGGGACCTTACC |
| *HPRT1* primers | Fwd: ACACTGGCAAAACAATGCAG  Rev: ACACTTCGTGGGGTCCTTTT |

**Zebrafish-MO details**

The designed sequences and primers used are described below:

| *GPX3*-MO: | TGAGTCCCCATGACTGTGTTTTTTA |
| --- | --- |
| *TNIP1*-MO: | CATCTGTCACCCGTCACTTACCAAA |
| MO control: | CCTCTTACCTCAGTTACAATTTATA |
| forward primer (Sequence 5’-3’): | GGGGacaagtttgtacAAAAAAgcaggctCAGGATCCCATATGGGcACgCAGAGCAACCCCTGGACCTC |
| reverse primer (Sequence 5’-3’): | GGGGaccacTTTGTAcaagaaagctgggtGGCTCGAGAGTTACTGTGCAGTCTGCAGAAG |

Design of the custom-GPX3 plasmid for rescue and overexpression

For the rescue and overexpression experiments, we designed a custom-*GPX3* mRNA (cst*GPX3*) with a modification of the first two codon following the ATG start codon (supplementary data) to protect the synthetic mRNA from being targeting by the *GPX3*-MO, while maintaining the same protein sequence. Initially, we amplified *danio rerio GPX3* using a forward primer including a gateway AttB1 and the aforementioned sequence modifications combined with a reverse primer including a gateway AttB2 site. The PCR amplicon obtained (**Supplementary Fig. S17**). was then recombined in pDONR221-P1-P2 using a gateway BP reaction, to generate a tol2-compatible clone (pME-dre_gpx3_Codon_optimised) (**Supplementary Fig. S18**). We then recombined this with tol2kit p3E302 (polyA) and a custom Destination clone for T3 RNA synthesis (pDEST-T3TS-R1-R3-For RNA expression, **Supplementary Fig. S19**) using a gateway multi-site LR(4-2-3) reaction. The resulting plasmid was named T3_dre_gpx3_Codon_optimised (**Supplementary Fig. S20**). To produce the cst*GPX3* mRNA used in this study, T3_dre_gpx3_Codon_optimised was then digested by *kpn1*, purified and used in MEGAscript™ T3 Transcription Kit reaction.

**Supplementary Figures:**

| 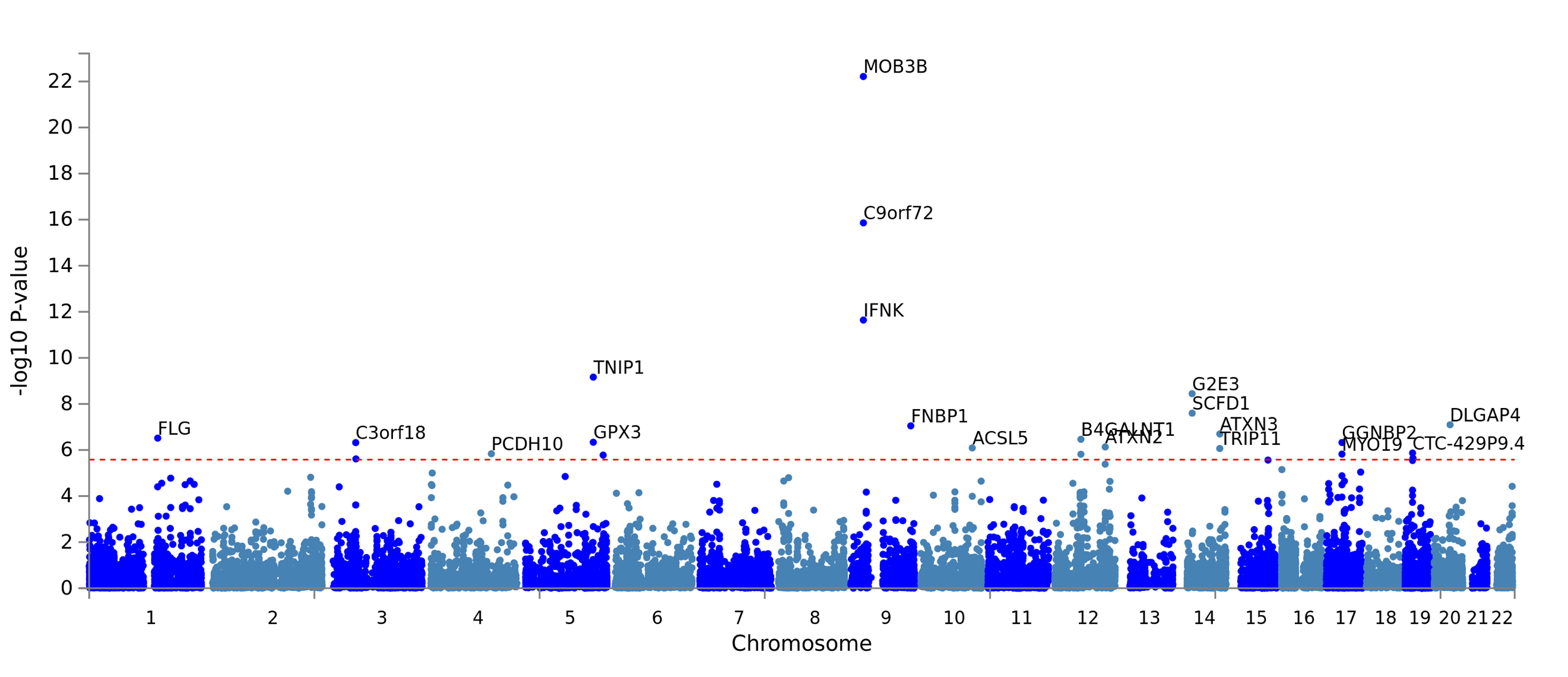  A. |
| --- |
| 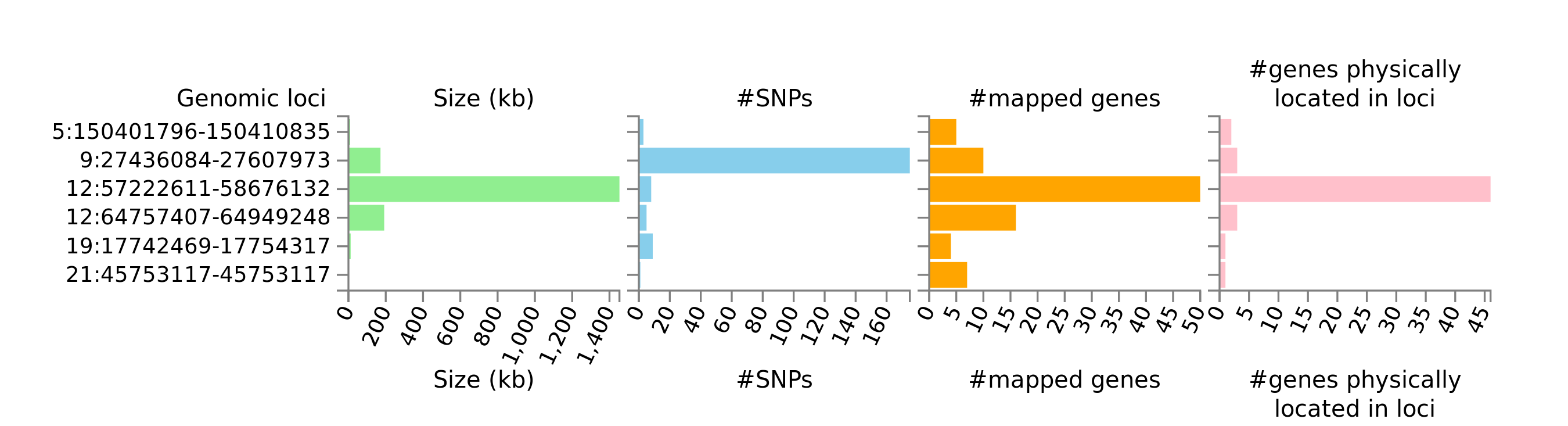  B. |
| 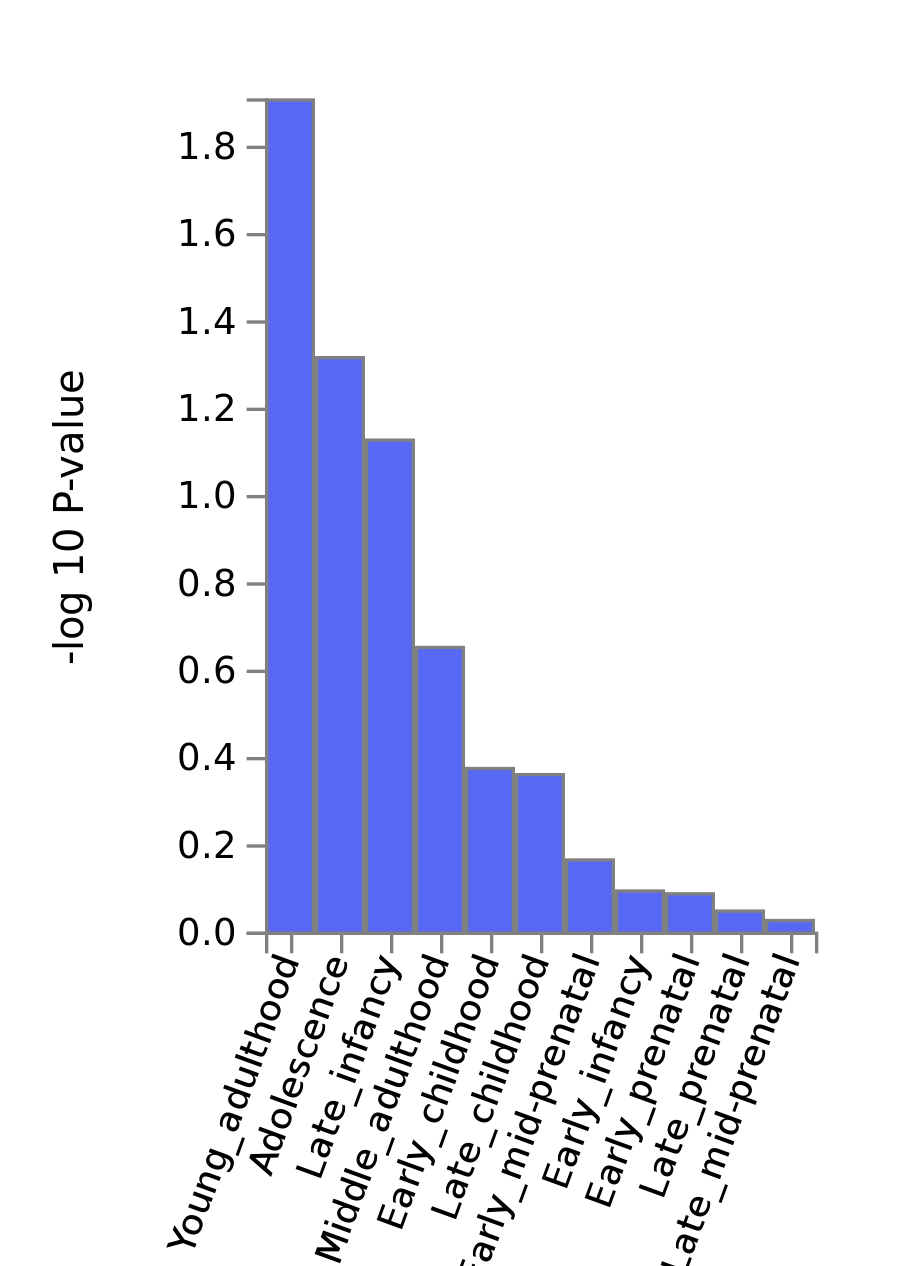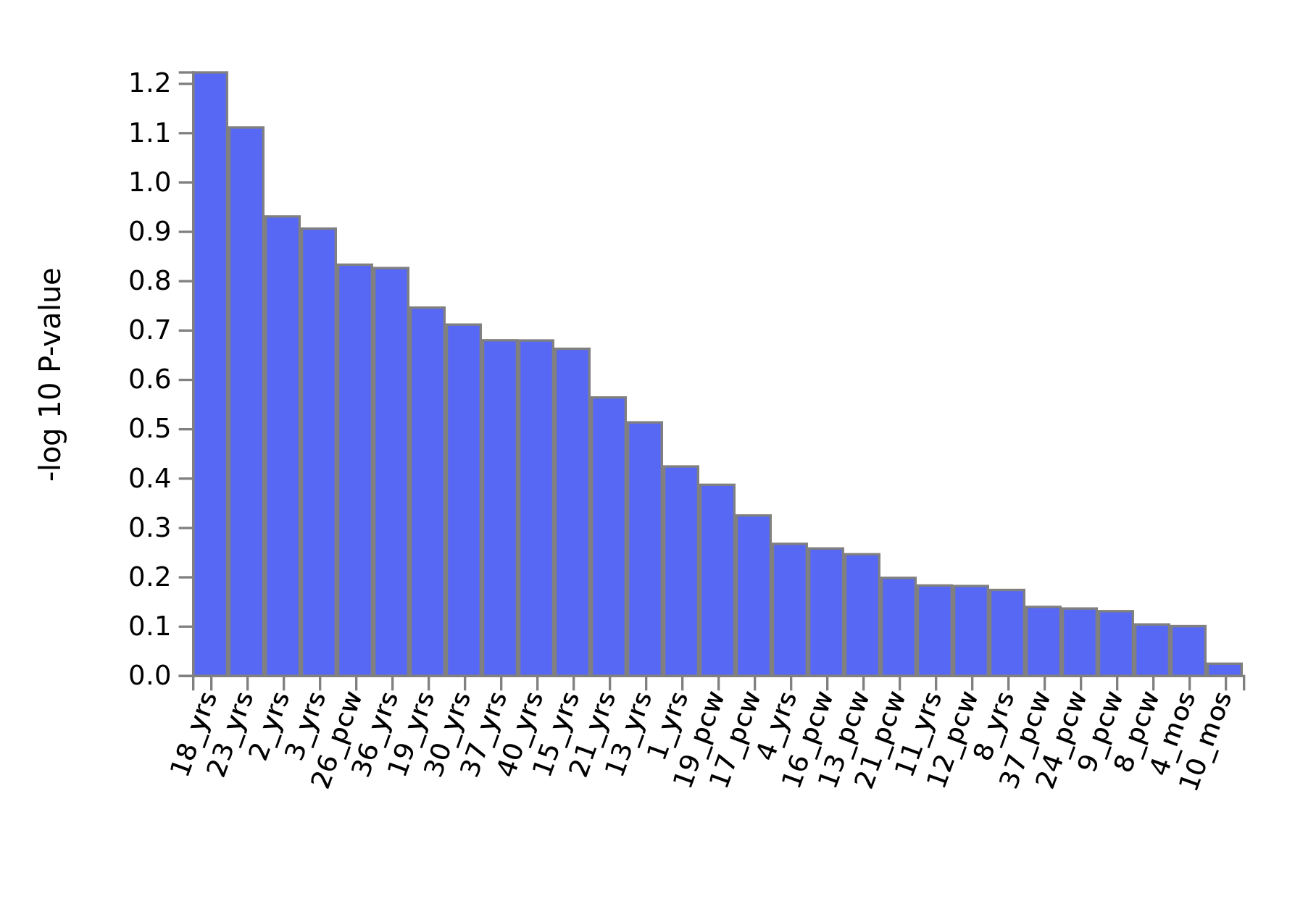  C. |

**Fig. S1. Functional Annotation and Mapping (FUMA) histogram summary** **A.** Manhattan plot of the gene-based test as computed by MAGMA based on GWAS summary statistics. The gene-based P-value is on the y-axis with significance threshold indicated in red (P < 2.6 x 10^-6^, (0.05/19071)), The top 24 genes are labelled. **B.** Six genomic loci were identified as significant, with size, number of SNPs, number of mapped genes and corresponding richness of genes location shown. **C.** BrainSpan 11 general developmental stages and 29 different ages of brain samples does not demonstrate a particular age of enrichment.

**
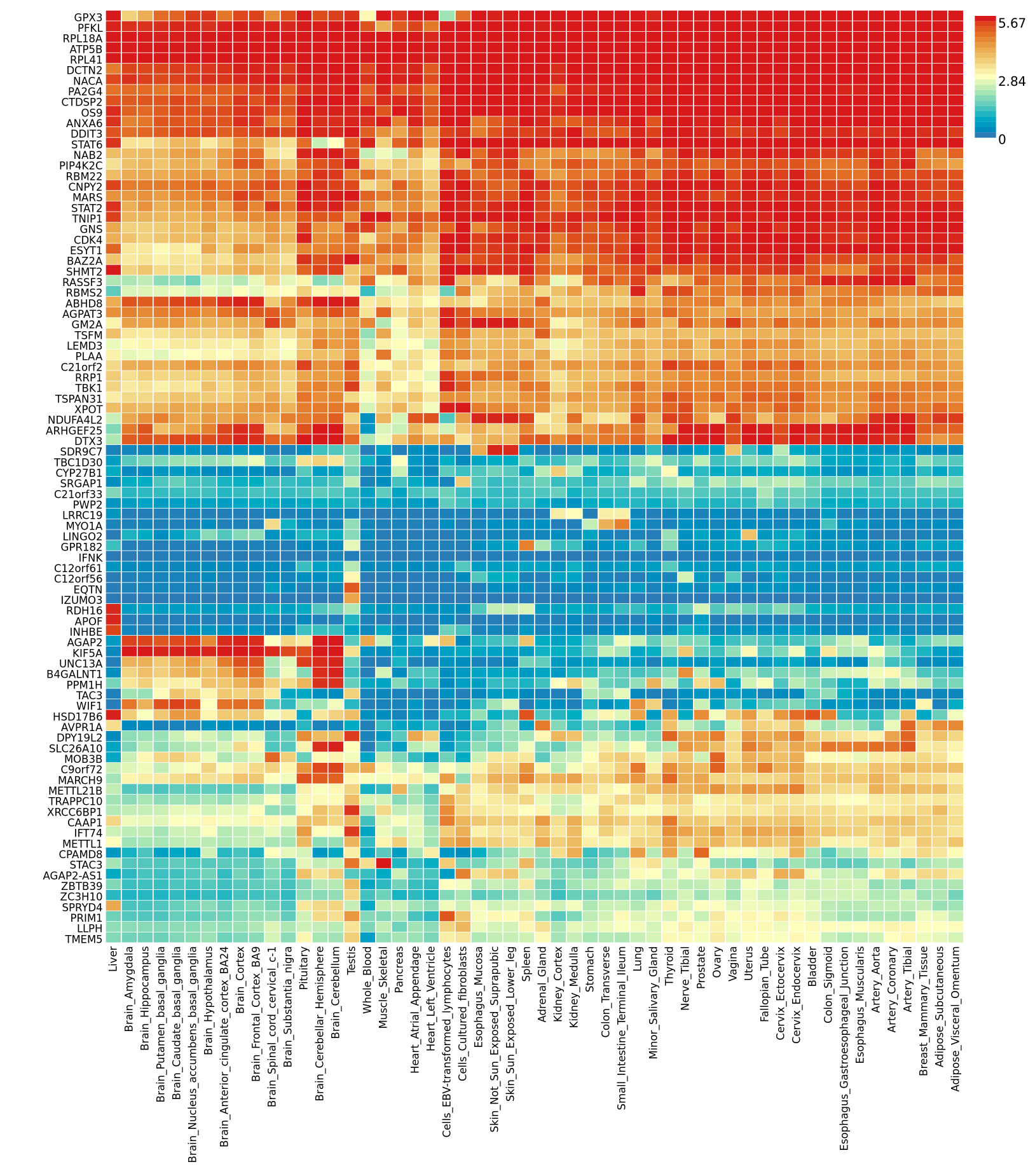
**

**Fig. S2. Functional Annotation and Mapping (FUMA) gene expression heat map.** Clustered Genes (average expression per label (log_2_ transformed) and tissues (GTEx v8 n=54) as computed by MAGMA based on GWAS summary statistics.

**B.**

**A.**

**Fig. S3. S-LDSC annotation enrichment in ALS GWAS A.** Results for all annotation categories **B.** Seven categories considered enriched (based on FDR correction of 0.05).

**Fig. S4. S-LDSC finds CNS and musculoskeletal cell-type categories are enriched in ALS GWAS**

| **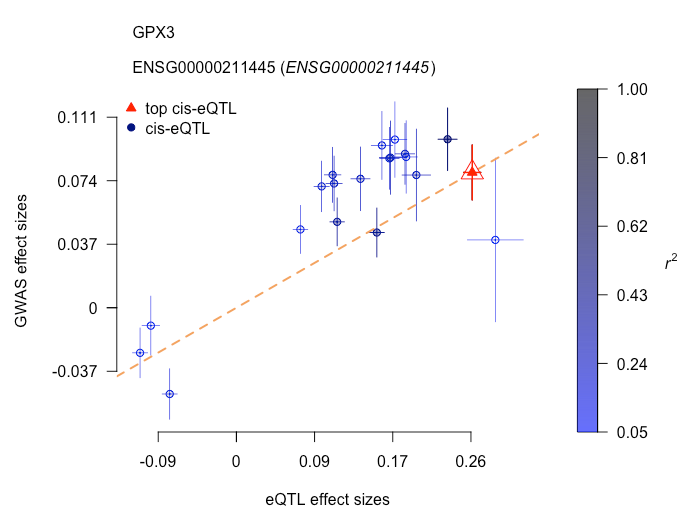A.** | **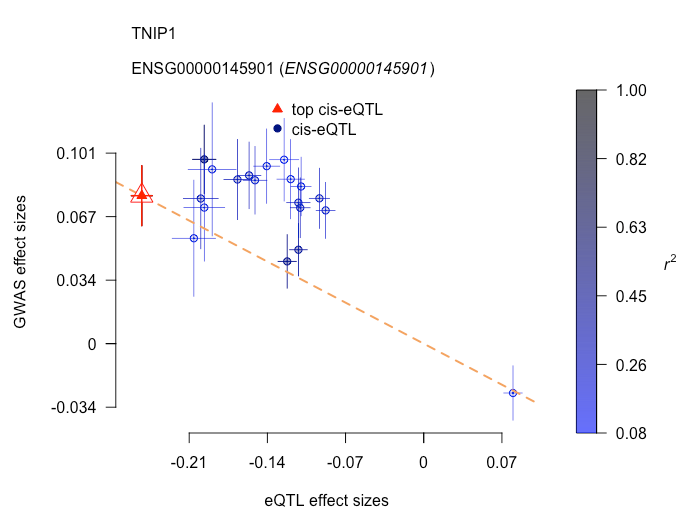B.** |
| --- | --- |
| **Fig. S5.** **Summary statistics-based Mendelian Randomization (SMR) analysis identifies *GPX3* an­d *TNIP1*** Association plot of A) GPX3 and B) TNIP1 from summary statistics-based Mendelian Randomization (SMR) analysis with the eQTL p-values of SNPs from the eQTLgen study for GPX3 and TNIP1. The plots include all the SNPs available in the region in the GWAS and eQTL summary data, respectively, rather than only the SNPs common to both data sets. Both genes have a significant SNP associated with ALS and gene expression levels of a probe within *GPX3* (p=1.05 x 10^-171^) and *TNIP1* (p=2.04 x 10^-163^) (eQTL) and the GWAS effect size are similar (both 0.08) however *TNIP1* B_SMR_effect_ size is -0.31 (negative) and *GPX3* has a (positive) eQTL B_SMR_effect_ of 0.30 (**Additional file 1: Table S12**). | |

**
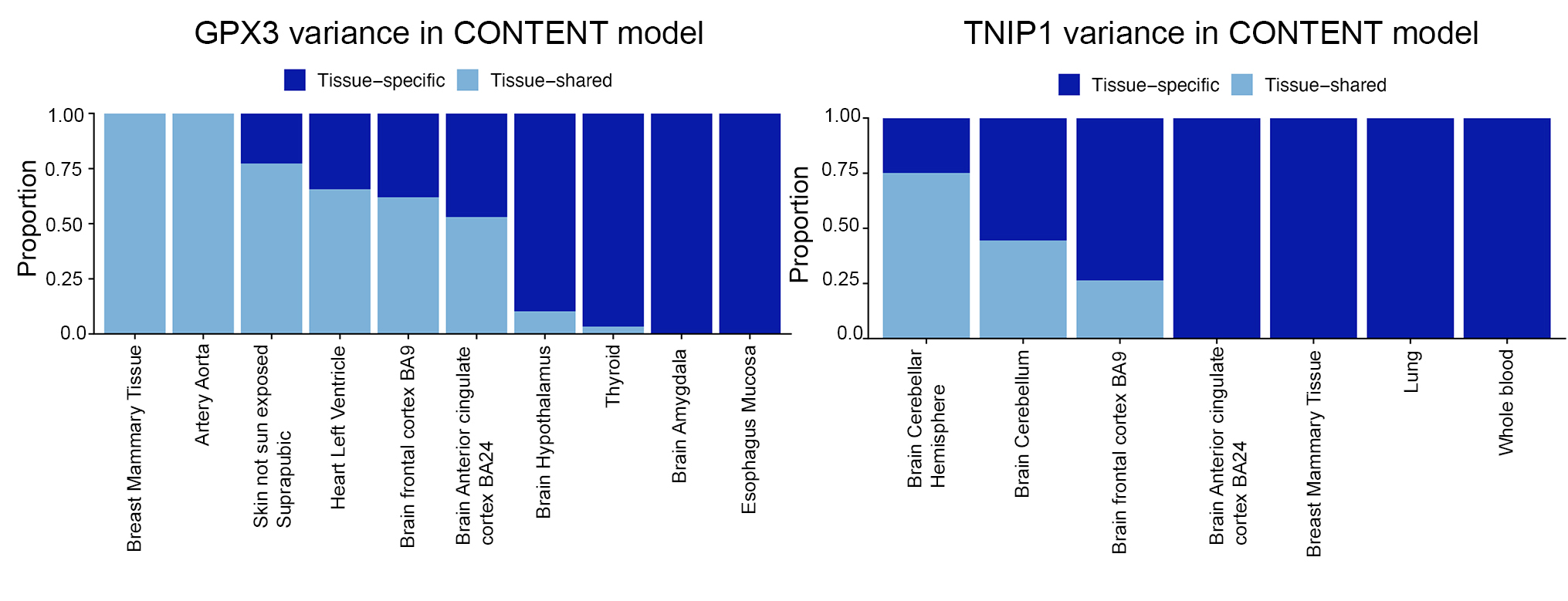
**

**Fig. S6.** **TWAS-CONTENT full model GPX3 and TNIP1 gene expression variance*.*** The proportion of tissue-specific expression and tissue-shared using TWAS-CONTENT full model. Tissues listed are those identified with significant expression of GPX3 and TNIP1 in the full model (*p*<0.0011, **Supplementary Table S18**) and the proportion of SNP-based heritability variance (0-1) associated with each tissue-shared and/or tissue-specific model.

| **A.**  **** |
| --- |
| **B.**  **** |
| **Fig. S7.** **Gene correlation across tissues** **A.** Correlation of expression across all tissues in GTEx shows a mean of 0.28±0.07 (similar to those or higher than with known ALS partners (**Supplementary Table S18**)) is no different to *GPX3* and *SOD1* (0.18±0.07) or *TNIP1* and *OPTN* (0.36±0.07). **B.** This correlation (dashed line) was above the median distribution of correlation across 16 genes that have been previously implicated with unequivocal evidence in ALS and ALS overlap syndromes) (Supplementary Table S19). |

| **** | **B.**  **** |
| --- | --- |
| **C.**  **** | **D.**  **** |
| **E.**  **** | **F.**  **** |
| **Fig. S8.** **Microarray expression data of *TNIP1* and *GPX3* demonstrate no difference in expression between cases and controls or genotype.** **A.** *TNIP1* expression in ALS cases and controls (*p* = 0.36) **B.** *GPX3* expression in ALS cases and controls **(***p* = 0.12) **C-D.** Association of additive model and recessive model with the rs10643311 risk allele in samples with matching genotype data. Additive and recession model *TNIP1:* *p*-value = 0.22 and 0.21 respectively. *GPX3:* additive model (*p*-value = 0.12), recessive model *p*-value = 0.02 **E-F**. Linear regression with surrogate variables, gender and site of onset as covariates found no correlation with age in controls. | |

| **A.**  **** | | **B.**  **** |
| --- | --- | --- |
| **C.**  **** | **D.** | |
| **Fig. S9.** **Preliminary Discovery cohort demonstrated association with GPX3 and ALS A.** No difference in level of GPX3 expression was detected in ALS cases compared to controls (n=50 Controls, n=50 ALS Cases) (*p*=0.43) **B.** Using genotyped cases and controls (n=89), there was no detected association with risk locus and GPX3 expression (*p* = 0.51). **C.** In ALS cases only, a positive association was detected with GPX3 and ALSFRS-R Score (*p* =0.0062, R^2^ = 0.16) (n=50) **D.** The direction was generally consistent with time since disease onset (i.e. lower GPX3 levels were associated with lower functional scores; association not significant, *p*=0.25, R^2^ = 0.017, n=49) (all analyses include sex as a covariate) | | |

| **A.** | **B.** |
| --- | --- |
| **C.** | **D.** |
| **E.** | |
| **Fig. S10.** **Replication GPX3 cohort** **A.** Male cases had a higher GPX3 level than female cases (626 ± standard error 249ng/ml, *p* = 0.013) **B.** There was no significant effect of age (n=195 cases, *p*=0.62) **C.** GPX3 was negatively associated with disease duration (time since onset of symptoms) (*p*=0.025, adjusted R^2^=0.040)^ **D.** GPX3 was associated with King’s Staging scale (p = 0.011, R^2^=0.040)^ **E.** Meta-analysis of GPX3 level and rs10463311 genotype in the discovery and replication cohorts indicated a trend for carriers of the ALS risk allele (C) to have lower levels of GPX3 (n=228 ALS cases, p = 0.08 additive model, p = 0.06 recessive (TT/TC vs CC) model). GPX3 levels (ng/ml) were standardised (mean of 0 and standard deviation of 1), sex was included as a covariate, all samples are independent (TT=137, TC=75, CC=16). Note: Retrospectively removing cases that did not meet the Gold Coast diagnostic criteria (n=10) resulted in no change to the association with GPX3 (n=188, *p*=1.0x10^-2^, R^2^=0.045, sex included as a covariate), additionally when we imputed missing values for rate progression using diagnosis or multiple visit data, the association of GPX3 with rate of progression was similar (n=190, *p*=4.9x10^-2^, R^2^=0.031, B_effect_ GPX3=-96±137 ng/ml/ALSFRS). ^= the effect size of these clinical variables and GPX3 was small, relative to the sex effect on GPX3 and thus sex could be driving these associations. | |

| **A.**  **** | **B.**  **** |
| --- | --- |
| **C.** | **D.**  **** |
| **Fig. S11.** **Preliminary longitudinal data assessment of ALSFRS-R and GPX3 levels** **A.** The change in ALSFRS-R in months since first visit identified a linear decrease of 0.54 points (0.42- 0.66 95% CI, p value = 7.2 x 10^-17^) **B.** The change in ALSFRS-R in months since diagnosis was 0.10 (0.034- 0.17 95% CI, p value = 2.8 x 10^-16^) (x-axis is limited to 50 to improve detail of graph for majority of cohort) **C.** The GPX3 level in months since first visit did not identify any common change across visits (-7.44 (-53.5 – 38.6 95% CI)) **D.** There was also no common change in GPX3 level across visits in months since diagnosis (-2.18 (-17.9 – 13.50 95% CI)) (For all results, n=89 individuals, total of 224 measurements, 1-5 visits) | |

| **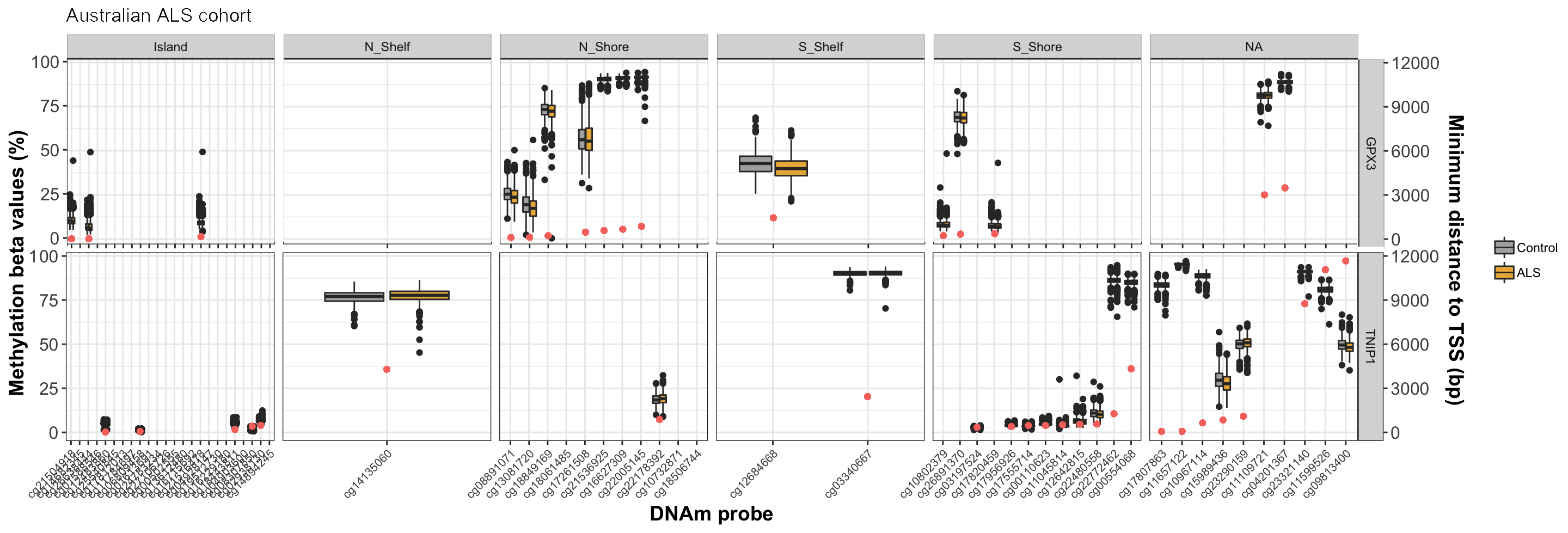** |
| --- |
| **Fig. S12. *TNIP1* and *GPX3* methylation in ALS and control blood** (*GPX3* (top row) and *TNIP1* (bottom row)). No changes in Beta methylation (%) were detected in probes in these genes between cases and controls. Additional visualisation of distance to the transcriptional start site (TSS) and functional regions (Cpg Island, Shelf, Shore and others) also did not reveal any relevant patterns of change. |

| **** |
| --- |
| **Fig. S13. Knockdown of *GPX3* and *TNIP1* in human motor neurons** qPCR analysis of **A.** *GPX3* and **B.** *TNIP1* expression levels in spinal motor neurons co-transfected with Hb9-GFP reporter constructs and target or scrambled siRNAs for 48 hr demonstrated validation of gene target knockdown in hESC-derived spinal motor neurons. Data represent mean ± SEM, n=3 independent transfections, *p<0.05, **p<0.01, (one-way ANOVA with Tukey's multiple comparison test) **C-D.** The hESC-derived spinal motor neuron viability normalised to Hb9-GFP only transfected cells (100%) (MTT reduction assay at 48 hr post co-transfection) mean±SEM, n=3 independent transfections, no statistical significance determined using two-way ANOVA with Sidak's multiple comparison test. **E-F.** Knockdown of *GPX3* and *TNIP1* had no effect on dominant neurite length or **G-H**) neurite and branch number |


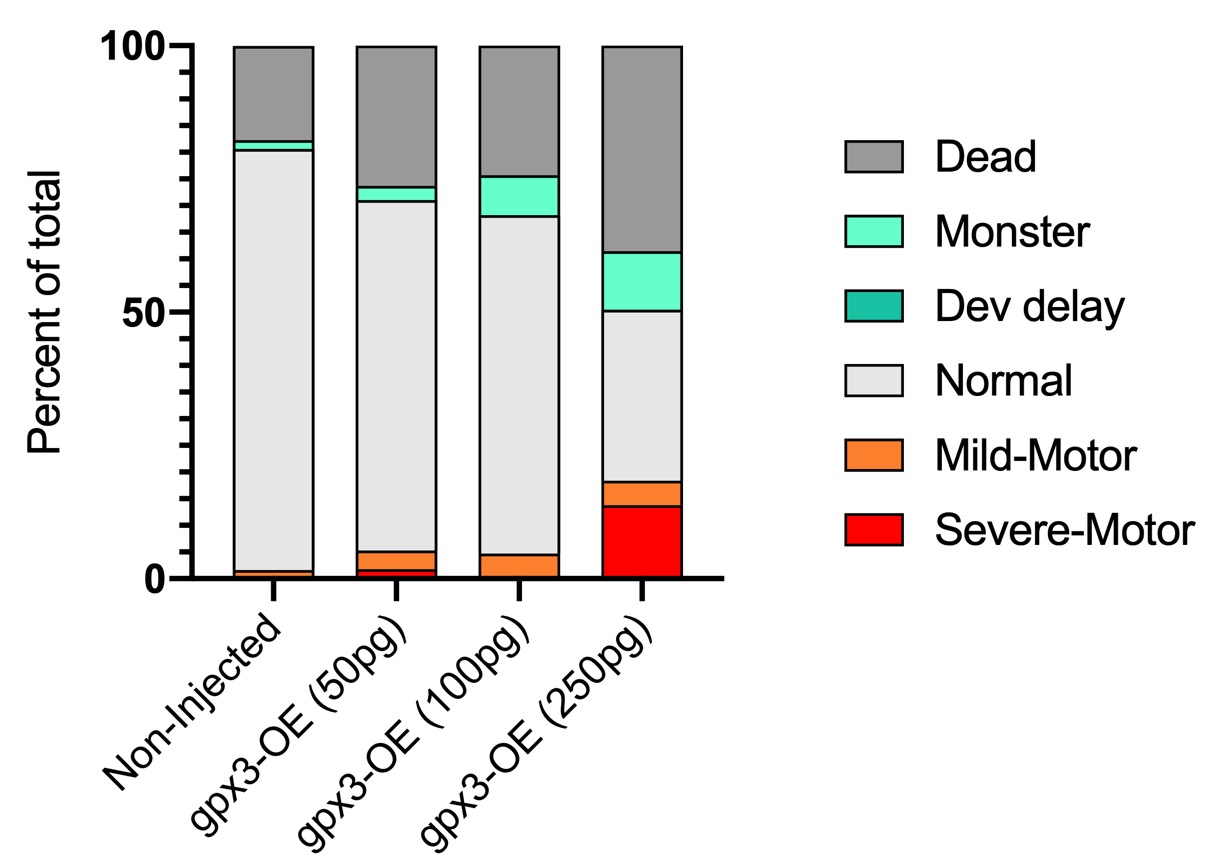


**Fig. S14. Motor and developmental effect of *GPX3* overexpression in zebrafish.** Zebrafish embryos were injected at the one-cell stage with cst-*gpx3* mRNA (*gpx3*-OE) at doses indicated. Morphological and behavioural analyses were conducted and did not indicate a motor-phenotype or obvious developmental effect at 50ng and 100ng, while toxicity evidenced by an increased number of malformed embryos (monsters) were reported at 250ng (**Additional file 1:Supplementary Table S24**). Due to the apparent toxicity at 250ng, a dose of 100ng has been selected for the LOF-rescue experiments.

**Fig. S15. Effect-size sensitivity analysis.** A sensitivity analysis of the effect-size on expression across different cohort sizes (minor allele frequency 0.24, alpha =0.05, power = 80%) demonstrates that with our sample sizes of n=200-1000 respectively, we were powered to detect an effect size of 0.032-0.015 (yi in SD units) (Rpackage = powerEQTL, function, powerEQTL.SLR)

**
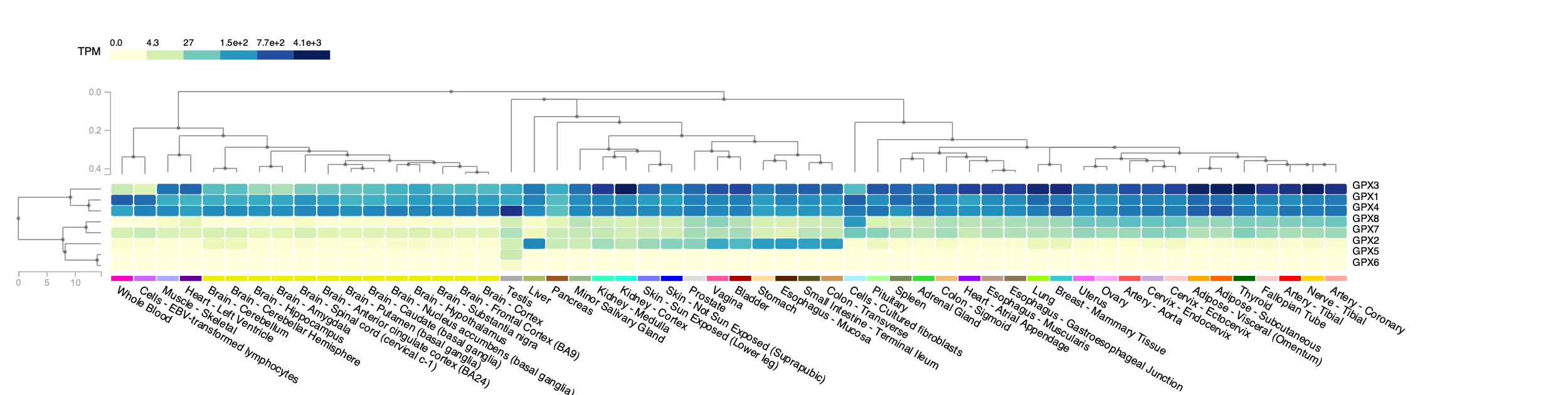
**

B.

A.

**
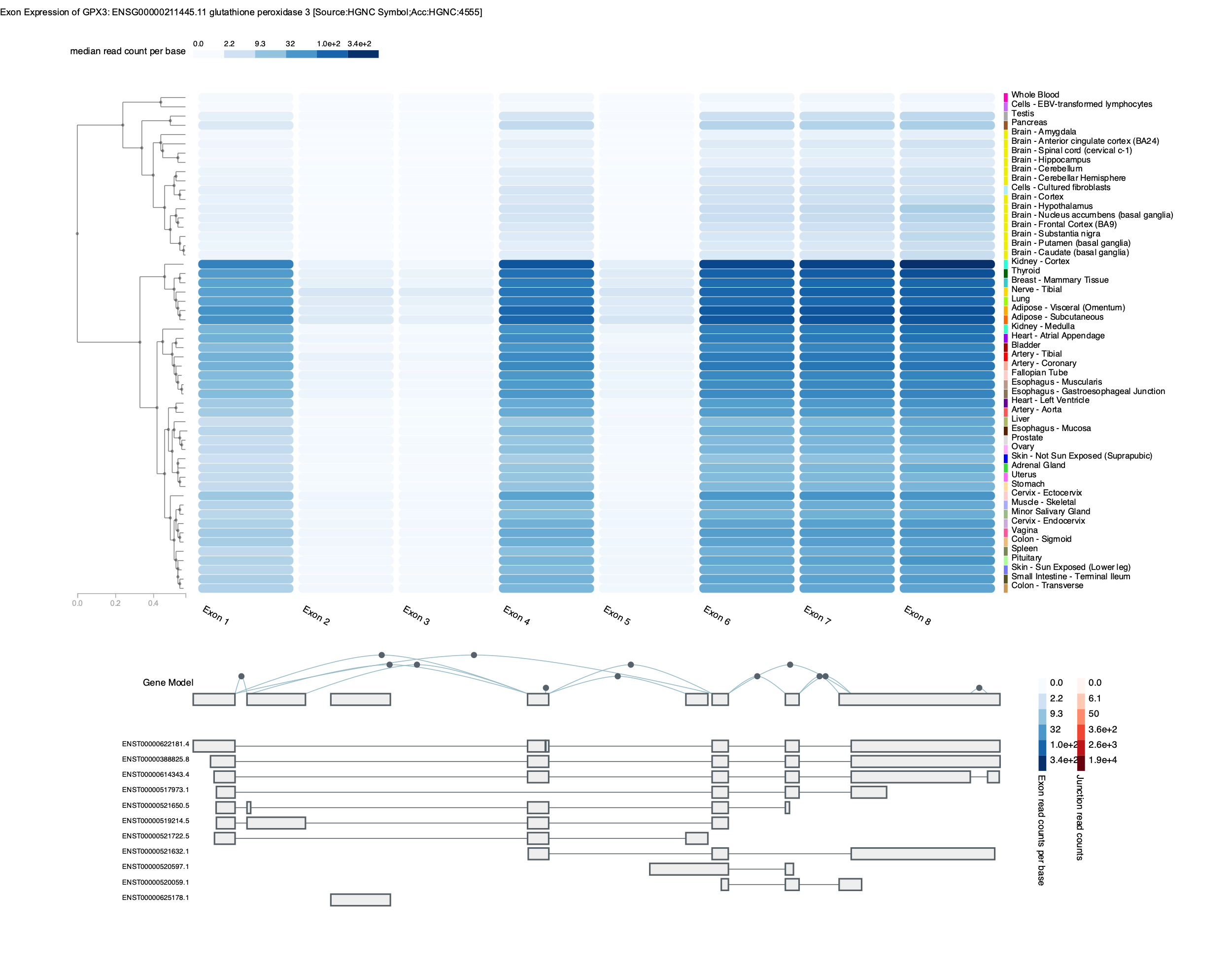
**

**Fig. S16. GPX and GPX3 expression pattern across tissues A.** GPX expression (*GPX1, GPX2, GPX3 GPX4, GPX5, GPX6, GPX7, GPX8*) clustered by tissues (GTEx v8, 54 tissues) demonstrates that GPX3 clusters with GPX1 and 4 and is highly expressed in most tissues with some variation in the brain and heart. Note: GPX3 is referred to as plasma GPX due to its high expression as a secreted protein (relative to other GPX family members), however this Fig. demonstrates the mRNA expression in whole blood and thus does not demonstrate this specific pattern in plasma. **B.** Eight exons are identified in the GPX3 gene, however higher expression of exons 1, 4, 6 and 8 exists in in most tissues.

** Fig. S17. Gpx3 amplicon for danio rerio.** Custom *gpx3*-mRNA generated for the rescue experiments. The first two codons were modified to protect the mRNA from being targeted by our anti-*gpx3*-MO. This sequence was cloned into a pME vector for straightforward downstream gateway recombination.

**Fig. S18. pME-dre_*gpx3*_Codon_optimised plasmid**. The custom made *gpx3* PCR amplicon was cloned into pDONR221-P1-P2 using a gateway BP reaction.

**Fig. S19. pDEST-T3TS-R1-R3-For RNA expression plasmid.** A custom destination plasmid for generating plasmids for T3 RNA synthesis.

**Fig. S20. T3_dre_gpx3_Codon_optimised plasmid.** The pME-dre_gpx3_Codon_optimised was recombined with tol2kit p3E302 (polyA) and the custom Destination clone for T3 RNA synthesis (pDEST-T3TS-R1-R3-For RNA expression) using a gateway multi-site LR(4-2-3) reaction. This plasmid was used to produce the cst-*gpx3*-mRNA used in this study for the rescue experiments.
